# Laminins in tumor-derived exosomes upregulated by ETS1 reprogram omental macrophages to promote omental metastasis of ovarian cancer

**DOI:** 10.1038/s41419-022-05472-7

**Published:** 2022-12-07

**Authors:** Haiyang Li, Cheng Zeng, Chang Shu, Yuanyuan Cao, Wengui Shao, Mengjie Zhang, Hongyong Cao, Shuli Zhao

**Affiliations:** 1 Department of General Surgery, Nanjing First Hospital, Nanjing Medical University, Nanjing, Jiangsu China; 2 General Clinical Research Center, Nanjing First Hospital, Nanjing Medical University, Nanjing, Jiangsu China; 3 General Clinical Research Center, Nanjing First Hospital, China Pharmaceutical University, Nanjing, Jiangsu China

**Keywords:** Cancer microenvironment, Ovarian cancer

## Abstract

Tumor-derived exosomes participate in omental metastatic colonization of ovarian cancer by inducing an adaptive response in the tumor microenvironment. However, cell–cell communication via exosomes between primary tumor cells and the microenvironment of distant omentum and the mechanism of pre-metastatic niche formation are poorly understood. Here, we demonstrated that ETS1-overexpressing ovarian cancer cells secreted larger exosomes with higher laminin levels. In addition, ovarian cancer exosomes could be taken up by omental macrophages through integrin and laminin interaction. Compared with control exosomes, exosomes derived from ETS1-overexpressing ovarian cancer cells (LV-ETS1 Exos) stimulated the polarization of more macrophages toward the M2 phenotype (CD163 marker), as well as the production of more CXCL5 and CCL2 in macrophages, via integrin αvβ5/AKT/Sp1 signaling. In vivo experiments showed that LV-ETS1 Exos promoted omental metastasis of ovarian cancer by mediating the tumor-promoting effect of macrophages, which could be neutralized by integrin ανβ5 inhibitor cilengitide. These results indicated that ETS1 could drive ovarian cancer cells to release exosomes with higher laminin levels, thereby accelerating the exosome-mediated pro-metastatic effects of omental macrophages via the integrin αvβ5/AKT/Sp1 signaling pathway, and the integrin ανβ5 inhibitor cilengitide could inhibit omental metastasis of ovarian cancer driven by tumor-derived exosomes.

## Introduction

Ovarian cancer was the leading cause of mortality among gynecological malignancies in 2020, with 313,959 new cases and 207,252 deaths globally [1]. Most patients with ovarian cancer have combined metastases at the time of diagnosis and have no evident clinical signs early on. Individuals with advanced ovarian cancer have a 5-year relative survival rate of only 29%; however, patients without metastases may have a survival rate of 92% [2]. Therefore, metastasis may be the leading cause of high mortality. In recent years, an increasing number of studies have revealed that the tumor microenvironment plays an important role in ovarian cancer metastasis [3–5]. However, the roles and mechanisms of the tumor microenvironment in ovarian cancer metastasis remain unclear.

The interaction between cancer cells and a particular microenvironment is essential for tumor metastasis [6]. Ovarian cancer metastasis is mostly implantation metastasis, with omental metastasis being the most frequent. Omental metastasis affects ~80% of patients with high-grade serous ovarian cancer [7, 8]. Stromal cells, including adipocytes, mesenchymal stem cells, fibroblasts, and macrophages, are predominant in the omentum [9, 10]. By secreting substances, such as lipids, cytokines, hormones, and exosomes, ovarian cancer cells drive the transformation of stromal cells into tumor-associated stromal cells, thereby forming a specific tumor microenvironment [11–13]. In this setting, various activated tumor-associated stromal cells co-aggregate with tumor cells to govern ovarian cancer metastasis [14]. According to various research findings reported in recent studies, exosomes enable paracrine communication between ovarian cancer cells and stromal cells in the microenvironment and may modify the normal extracellular matrix into an environment favorable for tumor cell development and metastasis [15, 16]. Exosomes are extracellular vesicles with a diameter of 50–140 nm that are released when the cell membrane fuses with the outer membrane of an intracellular multivesicular body. For intercellular signal transmission, they can transport a range of physiologically active small molecules, such as nucleic acids and proteins [17]. The synthesis, secretion, and contents of exosomes from tumor cells are controlled not only by the endosomal sorting complex required for transport mechanisms [18, 19] but also by tumor-specific signals such as DNA damage [20], oxidative stress [21] and p53 [22]. A study found that hypoxic pretreatment of CD34+ stem cells might boost the production of pro-angiogenic microRNAs in exosomes via ETS proto-oncogene 1 (ETS1) signaling [23]. ETS1 is an essential regulator of ovarian cancer metastasis [24], and its mRNA expression is related to the prognosis of patients with ovarian cancer [25]. Tomar et al. established a 3D culture model of ovarian cancer metastasis and discovered that ETS1 transcription in ovarian cancer cells was induced and activated by the microenvironment and that ETS1 can increase the expression of the downstream target FAK to promote cancer metastasis [26]. However, it remains unknown whether ETS1 can influence the metastasis of ovarian cancer by modulating exosome secretion and the content composition of tumor cells.

Therefore, we investigated the influence of ETS1 on tumor-derived exosomes secretion and content composition by regulating the expression of ETS1 in ovarian cancer cells and explored the impact of ETS1-mediated tumor-derived exosomes on omental metastasis in ovarian cancer. The clinical significance of ETS1 in omental metastasis and the prognosis of patients with ovarian cancer were retrospectively investigated. Our findings demonstrate that ETS1-overexpressing ovarian cancer cells can generate larger exosomes with higher laminin levels. These exosomes are more readily absorbed by omental macrophages, are able to drive the polarization of macrophages toward the M2 phenotype, and upregulate the expression of CXCL5 and CCL2 in macrophages, thereby boosting metastasis and omental colonization in ovarian cancer.

## Materials and methods

### Human tissue specimens and clinical data

According to cases with the diagnosis of ovarian cancer and uterine fibroid, a retrospective collection of 90 ovarian cancer specimens and uterine fibroid samples (*n* = 10) was conducted., from January 2013 to December 2020, at Nanjing First Hospital, Nanjing Medical University. These specimens were fixed in formalin and embedded in paraffin. Informed consent was obtained from all patients or their family members. This study complied with the requirements of the Ethics Committee of Nanjing First Hospital, Nanjing Medical University.

### Bioinformatics analyses of clinical data

Raw gene expression data for ovarian cancer (GSE178913, GSE26712, GSE18520, GSE105437, GSE14407, GSE10971, and GSE29450) and omental tissue (GSE122721 and GSE120196) were retrieved from the Gene Expression Omnibus (GEO) database. The Kaplan–Meier Plotter online analysis tool (https://kmplot.com/analysis/) was used to perform Kaplan–Meier analyses of patients with ovarian cancer from the GSE9891, GSE3149, GSE63885, GSE27651, GSE15622, and GSE30161 datasets. Correlation analyses of gene expression data and survival analysis from The Cancer Genome Atlas (TCGA) ovarian cancer cohort were conducted using the GEPIA analysis tool (http://gepia.cancer-pku.cn/).

### Tissue immunohistochemistry (IHC) and immunofluorescence (IF)

For IHC, human and murine tissues were fixed overnight in 4% paraformaldehyde, embedded in paraffin, and serially sectioned at 5-mm intervals. Endogenous peroxidase activity was blocked for 10 min using 3% hydrogen peroxide. The slides were submerged in 10 mM citrate buffer and microwaved for 15 min for antigen retrieval. Nonspecific binding was prevented for 10 min using 5% normal goat serum. Slides were incubated with various primary antibodies overnight at 4 °C in a humidified environment for 1 h at room temperature with the appropriate secondary antibodies. Hematoxylin was used as a counterstain after incubating sections with diaminobenzidine. For IF staining, slices were treated after antigen retrieval with a 10% goat serum-blocking solution at room temperature for 1 h. Tissue sections were incubated with primary antibodies overnight at 4 °C. After washing, the slices were incubated for 1 h at room temperature with Alexa Fluor-conjugated secondary antibody diluted in phosphate-buffered saline (PBS). The antibodies used for IHC, and IF are listed in Supplemental Table S2.

Tissue slices were assessed for IHC based on the degree and intensity of cell staining. The staining intensity was graded as 0 for negative, 1 for mild, 2 for moderate, and 3 for strong, and a proportion of cells that stained positively was assessed. Calculating the percentage of each part was done using the weighting method listed below (0–3): 0 × percentage of negative staining + 1 × percentage of light staining + 2 × percentage of moderate staining + 3 × percentage of intense staining. For IF, ImageJ was used to assess the fluorescence intensity quantification.

### Cell culture

The human ovarian cancer cell lines HO-8910 and A2780, the human monocytic cell line THP-1, and the murine monocytic cell line RAW 264.7 were obtained from the Type Culture Collection of the Chinese Academy of Sciences (Shanghai, China). HO-8910, A2780, and RAW 264.7 cells were cultured in Dulbecco’s modified Eagle medium (DMEM) supplemented with 10% fetal bovine serum (FBS) and 1% penicillin–streptomycin. THP-1 was cultured in RPMI 1640 cell culture medium containing 10% FBS and 1% penicillin–streptomycin, and cells were incubated with 100 ng/mL phorbol 12-myristate 13-acetate (PMA) for 24 h to induce differentiation into M0 macrophages. Short tandem repeat DNA profiling was used to identify all cell lines and check for mycoplasma contamination.

### Isolation of peritoneal macrophages

On day one, each mouse was injected intraperitoneally with 2 mL of 3% thioglycolate (Sigma-Aldrich, USA) and killed on day three. After injecting intraperitoneally 5 mL of RPMI 1640 cell culture medium with 10% FBS, penicillin, and streptomycin, peritoneal cells were collected in cell culture plates. Two hours later, the cells were washed with PBS to remove floating cells. The adherent cells were identified as peritoneal macrophages and subjected to further analysis.

### Cell transfection

The human ETS1 gene was amplified by PCR and cloned into a pcDNA vector to generate an ETS1 overexpression plasmid (pcDNA-ETS1). An empty vector plasmid (pcDNA-NC) was used as the negative control. All plasmids were obtained from KeyGene Biotech (Nanjing, China). siRNA oligonucleotides targeting ETS1 (siRNA: GAAUUACUCACUGAUAAATT) were designed and synthesized by KeyGene Biotech. Following the manufacturer’s instructions, Lipofectamine 3000 (Invitrogen, USA) reagent was used for plasmid and siRNA transfection.

Full-length ETS1 and GFP genes were inserted into GV502 vector to construct an ETS1 overexpression lentivirus (LV-ETS1). GFP lentivirus (LV-GFP) was used as the negative control. These lentiviruses were provided by GENECHEM (Shanghai, China). Target cells were transfected with LV-ETS1 or negative control LV-GFP and were subsequently treated with puromycin (2 μg/mL) for 2 weeks to generate stably transfected cells.

### RNA extraction and real-time quantitative polymerase chain reaction (qRT-PCR)

Following the manufacturer’s instructions, total RNA was extracted using TRIzol reagent (Invitrogen) and quantified using a NanoDrop spectrophotometer. Total RNA was reverse-transcribed using a cDNA reverse transcription kit (Takara, Japan). PCR amplification was performed at 95 °C for 30 s, followed by 40 cycles of 95 °C for 10 s and 60 °C for 34 s in a 7500 real-time PCR system with SYBR green (Bio-Rad, USA). GAPDH mRNA expression was used to normalize the target gene expression in each sample. Relative mRNA expression levels were analyzed using the 2^−ΔΔCt^ method. All primers were synthesized by Sangon Biotech (Shanghai, China). The primers used for qRT-PCR are listed in Supplemental Table S1.

### Protein extraction and western blotting

Proteins were extracted from cells and exosomes using RIPA buffer (KeyGene Biotech) containing a protease and phosphatase inhibitor (KeyGene Biotech), and the protein concentration was measured using a BCA Protein Assay Kit (KeyGene Biotech). A total of 10–30 μg of protein were loaded per well on an SDS-PAGE gel and transferred to a PVDF membrane (Millipore, USA). The membranes were rinsed with 1× TBST, blocked with 5% nonfat milk in 1× TBST for 2 h at room temperature, and then incubated with different primary antibodies. The next day, the membranes were washed three times with 1× TBST, incubated with the secondary antibody for 2 h, and then rinsed three times with 1× TBST. The signal was visualized using an ECL detection reagent (New Cell and Molecular Biotech, Suzhou, China) and a chemiluminescence device. The antibodies used for western blotting are listed in Supplemental Table S2.

### CCK-8 assay

Cell proliferation was determined using a CCK-8 kit, according to the manufacturer’s instructions (KeyGene Biotech). Briefly, 2000 cells/well were seeded into 96-well plates. CCK-8 detection solution (10 μl) was added to each well and incubated at 37 °C for 2 h. Optical density was measured at 450 nm.

### Colony-formation assay

A total of 1000 cells were placed into 60-mm dishes and then cultured in a medium with 10% FBS for 2 weeks. Colonies were washed with PBS, fixed in 4% paraformaldehyde for 15 min, and stained with crystal violet solution for 30 min. The numbers of colonies were counted using ImageJ software.

### Transwell assay

Transwell assays were used to determine cell migration and invasion abilities. For cell migration detection, 5 × 10^4^ cells were resuspended in 200 μl of serum-free medium. Then, the cells were seeded into the upper chamber of a Transwell assay insert (Corning, USA), and 650 μl 10% FBS medium was added to the lower chamber. After incubation at 37 °C for 48 h, the cells on the lower side were washed with PBS, fixed in 4% paraformaldehyde for 15 min, and stained with crystal violet solution for 30 min. Three random fields were chosen to count the stained cells for statistical analysis, and photographs were taken. For the invasion assay, Transwell chambers were coated with Matrigel (Corning) for 2 h at 37 °C. The cells (5 × 10^4^) were resuspended in 200 μl of serum-free medium and seeded into the upper chamber. Next, 650 μl 10% FBS medium was added to the lower chamber. After a 48-h incubation period, the invasive ability was evaluated as described above for the cell migration assay.

### Wound-healing assay

Cells were cultured in six-well plates. A straight scratch was made on the cell layer using a 200 μl tip across the center of the well. Cell debris was removed by washing twice with PBS, and cells were subsequently incubated with medium containing 2% FBS. Cell migration was monitored for 36 h in an incubator at 37 °C. The wounded area at the start and end of the experiment was measured using ImageJ software.

### Isolation and characterization of exosomes

For exosomes isolation, supernatant collected from ovarian cancer cells cultured in DMEM containing 10% Exosome-free FBS (System Biosciences, USA) for 48 h was centrifuged at 300 × *g* for 10 min, 3000 × *g* for 30 min and 10,000 × *g* for 30 min. Then, the supernatant was passed through 0.22-μm pore PES filters (Millipore). Ultracentrifugation was performed at 100,000 × *g* for 70 min using an Optima XE-90 Supercentrifuge (Beckman, Germany) to enrich the exosomes, and the pellet was rinsed using 30 ml of PBS. Finally, the exosomes were collected by ultracentrifugation at 100,000 × *g* for 70 min.

Isolated exosomes were mixed with 4% paraformaldehyde. exosomes were then dropped onto formvar carbon-coated electron microscopy grids and fixed with 1% glutaraldehyde for 10 min. Samples were negatively stained with 2% uranyl acetate solution. Images were obtained using Philips CM120 BioTwin transmission electron microscope (TEM) (FEI Company, USA).

Nanoparticle tracking analysis (NTA) was performed by Malvern Zetasizer Nano ZS-90 (Malvern Instruments Ltd., UK) following the manufacturer’s instructions. The exosomes were diluted in PBS. The mean particle size and size distribution were analyzed by dynamic light scattering method using the Malvern Zetasizer Nano ZS-90.

### Proteomic analysis

Total protein was extracted from exosomes and quantified. Label-free and LC-MS/MS analyses were performed by LC-Bio Technology (Hangzhou, China). Briefly, proteins were digested with trypsin at 37 °C overnight. The samples were separated using a NanoElute system (Bruker, Bremen, Germany) and analyzed by mass spectrometry using the PASEF mode of a timsTOF Pro mass spectrometer (Bruker) after separation. Proteomic data were processed using the label-free algorithm in MaxQuant. Proteins were filtered using human databases. Proteomic heatmaps were generated using the R composite heatmap package.

### Chromatin immunoprecipitation (ChIP) assay

ChIP assays were performed using a ChIP assay kit (Cell Signaling Technology, USA), according to the manufacturer’s instructions. Briefly, cells were cross-linked with 1% formaldehyde, quenched in glycine, lysed by sonication in the presence of protease inhibitors, and immunoprecipitated with anti-ETS1 antibody or nonspecific anti-IgG antibody (negative control; all Cell Signaling Technology). After washing, the DNA was released, eluted, qPCR-amplified, and the fragments were analyzed 2% using agarose gel electrophoresis. The primers used for ChIP-qPCR are listed in Supplemental Table S1.

### Exosome staining

To study exosome uptake, isolated exosomes were stained with PKH67 or PKH26 fluorescent dyes (Sigma-Aldrich, USA) according to the manufacturer’s protocol. Briefly, 4 μl of PKH67 or PKH26 was diluted in 500 μl of diluent C. Then, 100 μg of exosomes suspended in an equal volume of diluent C was added to the dye solution and incubated for 5 min at 37 °C. To stop the reaction, 1 ml FBS was added for 1 min. Subsequently, the stained exosomes were further centrifuged at 100,000×*g* for 70 min to remove excess dye. After discarding the supernatant, the stained exosomes were diluted with 100 μl of PBS and added to the cells. Following treatment for 12 h, the cells were washed with PBS and fixed with 4% paraformaldehyde for microscopic analysis.

### Co-culture of ovarian cancer cell-derived exosomes and macrophages

Exosomes were isolated from ovarian cancer cells that had been transfected with either LV-ETS1 or LV-GFP (LV-ETS1 Exos or LV-GFP Exos). Macrophages (derived from THP-1 cells or murine peritoneal macrophages) were seeded into 12-well plates, and exosomes were added to macrophages at a concentration of 10 μg/1 × 10^5^ cells, along with 1 μM cilengitide (MedChemExpress, USA) to co-culture for 48 h for subsequent experiments.

### Mouse studies

To investigate the metastatic effect of LV-ETS1 Exos in vivo, we established an omental metastasis model using 4-week-old female, specific pathogen-free BALB/c nude mice. Mice were randomly assigned to three different groups with five mice in each group: LV-ETS1 Exos group, LV-GFP Exos group and the LV-ETS1 Exos+ cilengitide group. All animal experiments were approved by the Animal Care Committee of the Nanjing First Hospital, Nanjing Medical University. Nude female mice were pre-educated with 10 μg exosomes resuspended in 100 μl PBS intraperitoneally injected every other day, along with 10 mg/kg cilengitide for three weeks, followed by an intraperitoneal injection of 5 × 10^6^ luciferase-positive A2780 (A2780-Luc) cells resuspended in 200 μl PBS. Four weeks after the intraperitoneal injection of A2780-Luc cells, tumor metastasis was detected using the IVIS Spectrum bioluminescence imaging system (PerkinElmer, USA). Five minutes before imaging, mice were injected with 10 μl/g body weight of 15 mg/ml D-luciferin potassium salt (Beyotime, Shanghai, China) dissolved in PBS. Finally, the mice were euthanized, and omental tumor samples were collected for flow cytometry and IHC analysis.

To assess the uptake of exosomes in the peritoneal cavity, PKH26- or PKH67-labeled exosomes were intraperitoneally injected into mice. After 24 h, ex vivo imaging of the major organs was performed using the IVIS Spectrum bioluminescence imaging system. For fluorescent antibody staining, a single-cell suspension was generated from mouse peritoneal omentum, and exosomes uptake by macrophages was detected using fluorescence microscopy or flow cytometry as described below.

### Single-cell preparation and flow cytometry

Murine omental tumor and omentum tissues were mechanically minced with scissors into small pieces, followed by digestion at 37 °C in a 5% CO_2_ incubator for 30 min in RPMI 1640 supplemented with 10% FBS, 1% penicillin–streptomycin, 0.05 mg/ml collagenase type I (Sigma-Aldrich), 0.025 mg/ml hyaluronidase (Sigma-Aldrich), and 0.01 mg/ml DNase I (Roche). Digested samples were passed through a 70-μm mesh, and red blood cells were lysed in RBC lysis buffer (Beyotime) for 3 min. Cell labeling was performed using fluorescently conjugated antibodies directed against mouse CD45, CD11b, F4/80, and CD163. Flow cytometry was performed on a DxFLEX flow cytometer (Beckman), and subsequent data were analyzed using FlowJo V10.

M0 macrophages derived from THP-1 cells and peritoneal macrophages were washed, resuspended, and stained with anti-CD163 antibody. Finally, the cells were analyzed using flow cytometry. The antibodies used for flow cytometry analyses are listed in Supplemental Table S3.

### Co-immunoprecipitation (Co-IP)

The cells were lysed with NP-40 lysis buffer (KeyGene Biotech) containing phosphatase and protease inhibitors. Cell lysates were incubated with individual antibody and protein A/G beads (MedChemExpress). After overnight incubation, the beads were washed four times with lysis buffer, separated by SDS-PAGE, and analyzed by immunoblotting. The antibodies used for immunoprecipitation are listed in Supplemental Table S2.

### Statistical analysis

Each experiment was performed in three independent replicates. Data are presented as the mean ± SD of the results. The two-sided Student’s *t* test was used for the comparison of two groups, and a parametric or nonparametric ANOVA test was used for multiple-comparison experiments. The association between ETS1 expression and clinicopathological variables of patients with ovarian cancer was statistically verified using Fisher’s exact test. All analyses were performed using GraphPad Prism 8 software (GraphPad Software). Survival curves were estimated using the Kaplan–Meier method and compared using the log-rank test. *P* values <0.05 were considered statistically significant. Where indicated, individual *P* values are shown; alternatively, the following symbols were used to describe statistical significance: *P < 0.05; **P < 0.01; ***P < 0.001; and ns, not significant.

## Results

### High ETS1 expression is closely related to omental metastasis and poor outcome in patients with ovarian cancer

**Fig. 1:**
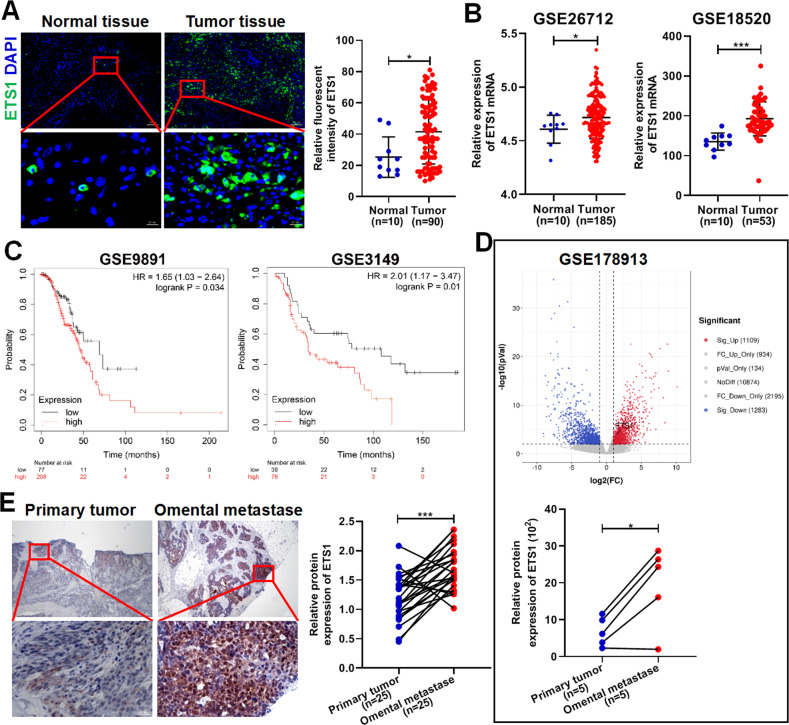
ETS1 is highly expressed in ovarian cancer omental metastases. **A** Representative immunofluorescence (IF) staining of ETS1 (green) and DAPI (blue) in ovarian cancer tissues and normal tissues. Scale bars: 100 μm at ×100 magnification; 20 μm at 630× magnification. **B** Expression of ETS1 mRNA in the ovarian cancer tissues and normal tissues from the GSE26712 and GSE18520 datasets. **C** Kaplan–Meier plots showing the overall survival of ovarian cancer patients from the GSE9891 and GSE3149 datasets based on ETS1 mRNA expression. **D** Volcano plot showing the differentially expressed genes between five pairs of the ovarian primary tumor and corresponding omental metastases, and the bottom graph showing the expression of ETS1 mRNA in the ovarian primary tumor and corresponding omental metastases from the GSE178913 database. **E** Representative immunohistochemistry (IHC) staining of ETS1 in ovarian primary tumors and corresponding omental metastases. Scale bars: 200 μm at ×40 magnification; 20 μm at ×400 magnification. Data are shown as mean ± SD. *P < 0.05, ***P < 0.001.

To clarify the clinical significance of ETS1 in ovarian cancer, we investigated the correlation between ETS1 expression and clinicopathological characteristics in 90 ovarian cancer samples. Compared to the normal ovarian surface epithelium collected from patients with uterine fibroids, ETS1 expression in tumor tissue was found to be higher (Fig. 1A). By integrating the GSE26712, GSE18520, GSE105437, GSE14407, and other datasets, we further confirmed that ETS1 expression was higher in ovarian cancer tissues than in normal tissues (Fig. 1B and Supplementary Fig. S1). Fisher’s exact test of ETS1 expression and clinicopathological characteristics revealed that ETS1 expression was associated with tumor size and distant metastasis status but not with lymph node metastasis status and histological differentiation (Table 1). In addition, the survival analysis of cases extracted from the GSE9891, GSE3149, and GSE63885 showed that ETS1 expression was positively associated with poor prognosis in patients with ovarian cancer (Fig. 1C and Supplementary Fig. S2A), however, the survival analysis in the GSE27651, GSE15622, GSE30161, and TCGA ovarian cancer cohorts did not reach statistical significance (Supplementary Fig. S2B–E).

**Table 1:** Correlation between ETS1 expression and clinicopathological characteristics in 90 ovarian cancer patients.

Clinicopathological variables	ETS1 expression		*P* value
	Low (*n* = 38)	High (*n* = 52)	
Age (years)			
<55	22	28	0.8304
≥55	16	24	
Grade			
G1 + G2	14	30	0.0580
G3	24	22	
T classification			
T1 + T2	18	13	0.0426
T3	20	39	
N classification			
N0	21	23	0.3936
N1	17	29	
M classification			
M0	25	20	0.0184
M1	13	32	

To confirm the significance of ETS1 expression for omental metastasis of ovarian cancer, we analyzed differentially expressed genes in the GSE178913 database, including five pairs of primary tumors and their corresponding omental metastases. We found that the expression of ETS1 mRNA in omental metastases was significantly higher than that in primary tumors (Fig. 1D). IHC staining confirmed that ETS1 expression was higher in omental metastases (Fig. 1E).

### ETS1 promotes ovarian cancer cell proliferation, migration, and invasion in vitro

**Fig. 2:**
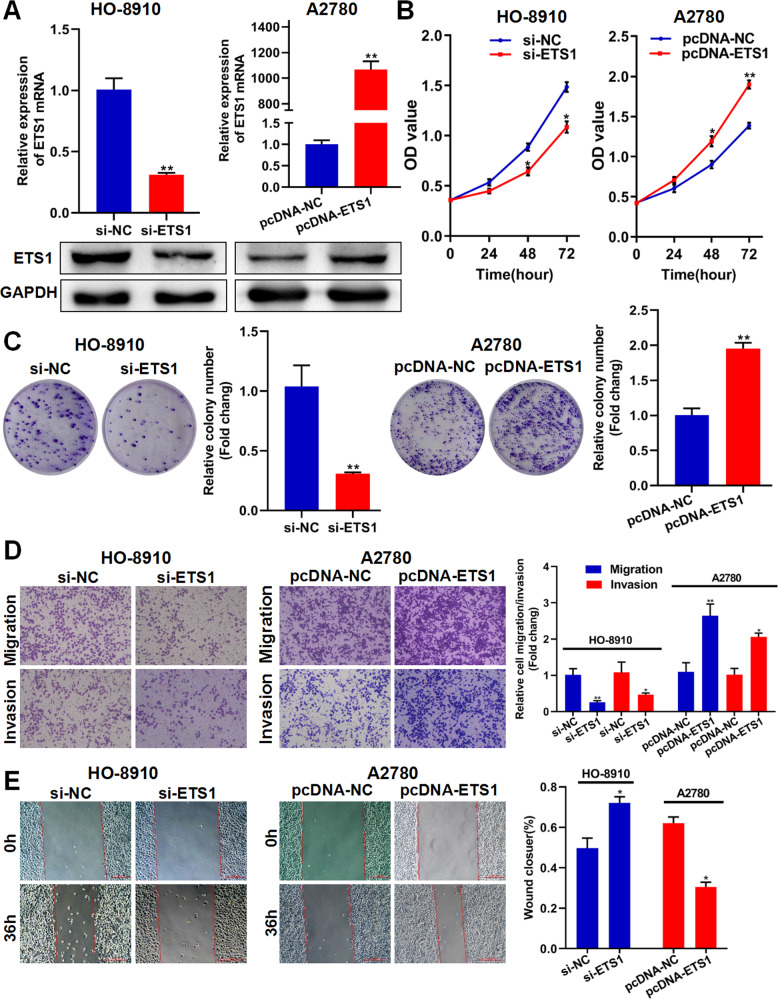
ETS1 promotes ovarian cancer cell proliferation, migration, and invasion in vitro. **A** The validation of knockdown and overexpression efficacy of ETS1 in ovarian cancer cells by qRT-PCR and western blotting. **B** CCK-8 assay showing the effect of ETS1 knockdown and overexpression on proliferation in ovarian cancer cells. **C** Effects of ETS1 knockdown and overexpression on colony formation in ovarian cancer cells. **D** Effects of ETS1 knockdown and overexpression on migration and invasion in ovarian cancer cells. **E** Wound-healing assay showing the horizontal migration ability with ETS1 knockdown or overexpression in ovarian cancer cells. Data are shown as mean ± SD. *P < 0.05, **P < 0.01.

To determine the role of ETS1 in the progression of ovarian cancer, si-ETS1 and pcDNA-ETS1 were used to knock down or overexpress ETS1 in HO-8910 and A2780 cells, respectively. As shown in Fig. 2A, si-ETS1 and pcDNA-ETS1 efficiently knocked down and overexpressed ETS1 in ovarian cancer cells, respectively. Colony formation and CCK-8 assays demonstrated that knockdown of ETS1 inhibited the proliferative capacity of HO-8910 cells, whereas overexpression of ETS1 promoted the proliferative capacity of A2780 cells (Fig. 2B, C). Using the Transwell assay, we discovered that ETS1 downregulation greatly decreased ovarian cancer cell migration and invasion capacity, whereas ETS1 overexpression enhanced ovarian cancer cell migration and invasion (Fig. 2D). The wound-healing assay demonstrated that ETS1 facilitated the migration of ovarian cancer cells (Fig. 2E). These findings imply that ETS1 plays a crucial role in ovarian cancer cell proliferation, migration, and invasion.

### ETS1-overexpressing ovarian cancer cells generate larger-sized exosomes with higher laminin levels

**Fig. 3:**
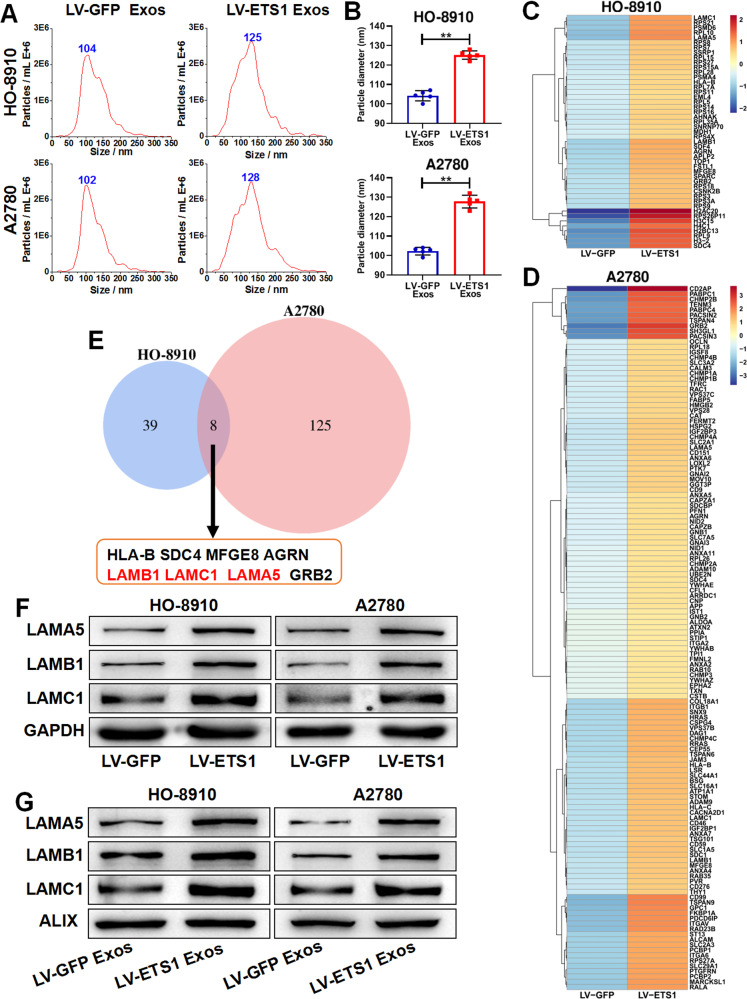
ETS1-overexpressed ovarian cancer cells secrete larger-sized exosomes with higher laminin levels. **A** Concentration and size distribution of exosomes derived from ovarian cancer cells transfected with lentivirus LV-ETS1 and LV-GFP (LV-ETS1 Exos and LV-GFP Exos) calculated by nanoparticle tracking analysis (NTA). **B** Average diameter of LV-ETS1 Exos and LV-GFP Exos. **C**, **D** Heatmap diagrams of upregulated protein between LV-ETS1 Exos and LV-GFP Exos derived from HO-8910 (**C**) and A2780 cells (**D**). Protein expression data were obtained using Label-free. **E** Venn diagram showing eight proteins that were upregulated in both cell lines LV-ETS1 Exos. **F** Western blotting showing LAMA5, LAMB1 and LAMC1 protein expression in ovarian cancer cells transfected with lentivirus LV-ETS1 and LV-GFP. **G** Western blotting showing LAMA5, LAMB1 and LAMC1 protein expression in LV-ETS1 Exos and LV-GFP Exos derived from HO-8910 and A2780 cells. Data are shown as mean ± SD. **P < 0.01.

To investigate the influence of ETS1 on the secretion and composition of exosomes generated from ovarian cancer cells, we overexpressed ETS1 in ovarian cancer cells using the lentiviral vector LV-ETS1. ETS1 expression levels of HO-8910 and A2780 cells were assessed using qRT-PCR and western blotting (Supplementary Fig. S3A). Exosomes released from stably ETS1-overexpressing ovarian cancer cells were collected for further research. TEM and NTA analyses of LV-ETS1 Exos and LV-GFP Exos confirmed that the isolated particles displayed the characteristic size and morphology of exosomes (Supplementary Fig. S3B and Fig. 3A). In addition, we observed discrepancies in size distribution between LV-ETS1 Exos and LV-GFP Exos. More than 90% of LV-GFP Exos were around 100 nm in diameter, whereas the majority of LV-ETS1 Exos were ~125 nm in diameter (Fig. 3B). The significant difference in particle size between LV-ETS1 Exos and LV-GFP Exos indicated possible changes in their compositional contents. However, the number of exosomes released by each LV-ETS1 cell was not substantially different from that released by each LV-GFP cell (Supplementary Fig. S3C). To further corroborate the features of the isolated exosomes, we identified LV-ETS1 Exos and LV-GFP Exos with exosome surface markers using western blotting (Supplementary Fig. S3D).

Proteins are important components of exosomes. We collected LV-ETS1 Exos and LV-GFP Exos from HO-8910 and A2780 cells and extracted total protein from these exosomes for proteomic analysis. Comparing the protein expression profiles of LV-ETS1 Exos and LV-GFP Exos, we found that 47 and 133 proteins were significantly upregulated in LV-ETS1 Exos from HO-8910 and A2780 cells, respectively (Fig. 3C, D). Further analyses revealed that eight proteins (LAMA5, LAMB1, MFGE8, AGRN, LAMC1, GRB2, HLA-B, and SDC4; Fig. 3E) in LV-ETS1 Exos were significantly upregulated in both cell lines. Among these proteins, LAMA5, LAMB1, and LAMC1 belong to the laminin family. Laminin is involved in tumor infiltration and metastasis and is associated with poor tumor prognosis [27]. LAMA5, LAMC1, and LAMB1 were also the proteins with the highest fold increase among the eight proteins, except for MFGE8; therefore, we focused on LAMA5, LAMB1, and LAMC1 in follow-up studies. Western blotting confirmed that the protein expression levels of LAMA5, LAMC1, and LAMB1 in A2780 and HO-8910 LV-ETS1 cells and their exosomes were higher than those in corresponding LV-GFP control cells (Fig. 3F, G).

In ovarian cancer samples from the TCGA database, the expression levels of LAMA5, LAMB1, and LAMC1 were positively correlated with that of ETS1 (Supplementary Fig. S4A). In a survival analysis of patients with ovarian cancer in the GSE9891 dataset, increased expression of LAMA5, LAMB1, and LAMC1 was related to shorter overall survival (Supplementary Fig. S4B). We determined the expression of these three genes in ETS1-knockdown HO-8910 cells to better understand the regulatory function of ETS1 on LAMA5, LAMB1, and LAMC1 expression in ovarian cancer. According to qRT-PCR and western blotting data, ETS1 regulated the expression of LAMA5, LAMB1, and LAMC1 in ovarian cancer cells (Supplementary Fig. S4C, D). Furthermore, we extracted exosomes from the cell supernatants of ETS1-knockdown HO-8910 cells and control cells. We found that the protein expression level of LAMA5, LAMB1, and LAMC1 in the exosomes of the ETS1-knockdown group were downregulated (Supplementary Fig. S4E). We also identified putative binding sites for ETS1 in the promoter regions of LAMA5, LAMB1, and LAMC1 using the Jaspar^2022^ database (Supplementary Fig. S4F). ChIP was performed on nuclear extracts from HO-8910 cells using an anti-ETS1 antibody. The results showed that the promoter regions of LAMA5, LAMB1, and LAMC1 were enriched in immunoprecipitates with anti-ETS1 antibodies (Supplementary Fig. S4G). The results show that ETS1 transcriptionally regulates the expression of LAMA5, LAMB1, and LAMC1 in ovarian cancer cells.

### ETS1 promotes the uptake of ovarian cancer exosomes by omental macrophages

**Fig. 4:**
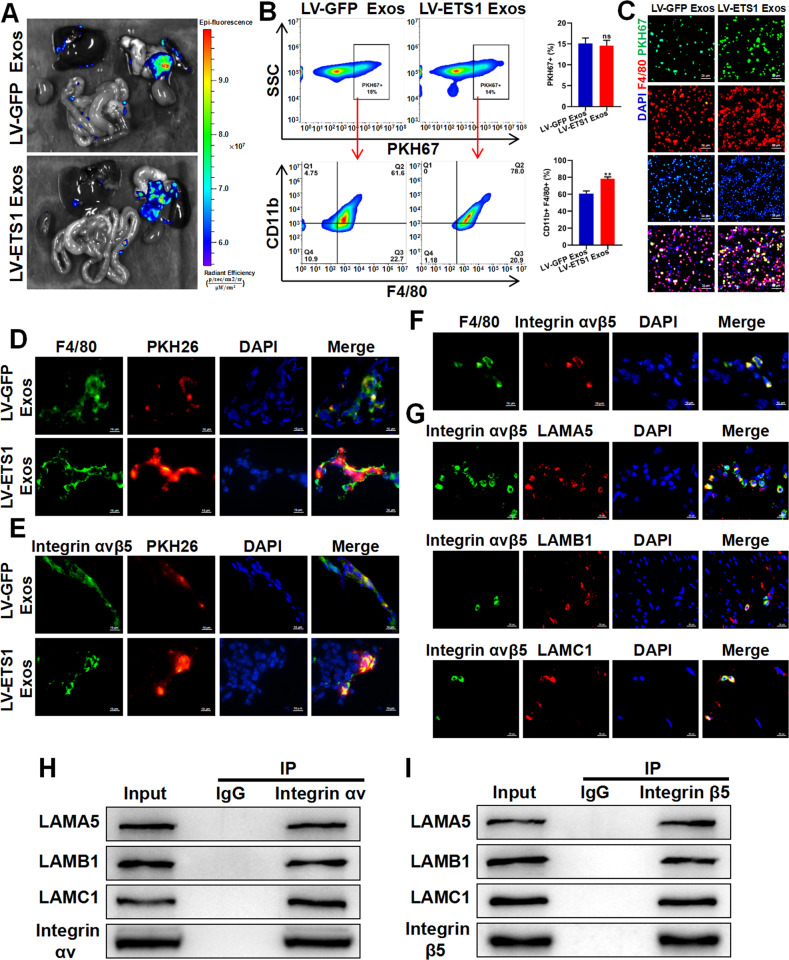
ETS1 promotes ovarian cancer exosomes uptake by omental macrophages. **A** Ex vivo bioluminescence analysis showing uptake of ovarian cancer exosomes by abdominal organs. **B**, **C** Flow cytometry analysis (**B**) and fluorescence microscope observation (**C**) showing uptake of PKH67-labeled LV-ETS1 Exos and LV-GFP Exos by omental macrophages. Scale bars: 20 μm at ×200 magnification. **D** Immunofluorescence analysis showing PKH26-labeled LV-ETS1 Exos and LV-GFP Exos (red), F4/80 (green), and DAPI (blue) staining in nude mice omentum. Scale bars: 10 μm at ×630 magnification. **E** Immunofluorescence analysis showing PKH26-labeled LV-ETS1 Exos and LV-GFP Exos (red), integrin αvβ5 (green) and DAPI (blue) staining in nude mice omentum. Scale bars: 10 μm at ×630 magnification. **F** Immunofluorescence analysis showing F4/80 (green), integrin αvβ5 (red) and DAPI (blue) staining in nude mice omentum. Scale bars: 10 μm at ×630 magnification. **G** Immunofluorescence analysis showed LAMA5, LAMB1 and LAMC1 (red) colocalized with integrin αvβ5 (green) in nude mice omentum. Scale bars: 10 μm at ×630 magnification. **H**, **I** Co-IP analysis showed LAMA5, LAMB1 and LAMC1 could interact with integrin αv (**H**) and integrin β5 (**I**) protein in RAW 264.7 cells. Data are shown as mean ± SD. **P < 0.01, ns non-significant.

Next, we investigated the dispersal of ovarian cancer exosomes in abdominal organs in vivo. We injected 10 μg of PKH67-labeled LV-ETS1 Exos and LV-GFP Exos derived from A2780 cells into the abdominal cavity of nude mice and observed the biodistribution and uptake of exosomes in abdominal organs 24 h after injection. We observed that LV-ETS1 Exos and LV-GFP Exos were mainly enriched in the omentum (Fig. 4A). To further investigate which cells in the omentum mainly take up the exosomes, we removed the omentum after exosome uptake and made single-cell suspensions for flow cytometry and fluorescence microscopy analyses. Flow cytometry results showed that major exosomes were taken up by omental CD11b + F4/80+ macrophages, and the proportion of LV-ETS1 Exos taken up by CD11b + F4/80+ macrophages was higher (Fig. 4B). The results of the fluorescence microscopy analysis of single-cell suspensions showed that F4/80+ macrophages took up more LV-ETS1 Exos (Fig. 4C). These findings indicate that ovarian cancer exosomes are primarily ingested by omental macrophages and that ETS1 can facilitate this process.

Next, we investigated why ETS1 induced the uptake of ovarian cancer exosomes by omental macrophages. The laminin family members LAMA5, LAMB1, and LAMC1 were the most prominently upregulated proteins in LV-ETS1 Exos of the two ovarian cancer cell lines. Several integrin family members function as laminin receptors in various cell types [28]. Therefore, we evaluated the mRNA expression of integrin family genes in the omentum by integrating omentum-related data from the GSE120196 and GSE122721 datasets (Supplementary Fig. S5A, B). The integrins αv and β5 were highly expressed in both databases; therefore, we decided to focus our study on these two integrins. According to IHC results, the expression levels of integrin αv and integrin β5 in omental tissues from ovarian cancer patients with omental metastases were greater than those from individuals with benign gynecological illness (Supplementary Fig. S5C, D). As shown in Fig. 4D, E, we found exosomes preferentially colocalized with integrin αvβ5-positive cells and F4/80+ macrophages in the omentum, and LV-ETS1 Exos were more readily assimilated by F4/80+ macrophages and integrin αvβ5-positive cells than LV-GFP Exos. Moreover, integrin αvβ5 was present in omental F4/80+ macrophages (Fig. 4F). To verify the interaction of LAMA5, LAMB1, and LAMC1 with integrin αvβ5, we performed Co-IP and immunofluorescence colocalization assays. Immunofluorescence showed that LAMA5, LAMB1, and LAMC1 colocalized with integrin αvβ5 in the omentum (Fig. 4G). The Co-IP assay demonstrated that LAMA5, LAMB1, and LAMC1 were capable of binding to integrin αvβ5 (Fig. 4H, I). The above data suggested that the greater uptake of LV-ETS1 Exos by omental macrophages was due to the interaction of higher laminin levels in LV-ETS1 Exos and integrin αvβ5.

### LV-ETS1 Exos induce macrophage polarization toward M2 phenotype and increase CXCL5 and CCL2 expression in macrophages

**Fig. 5:**
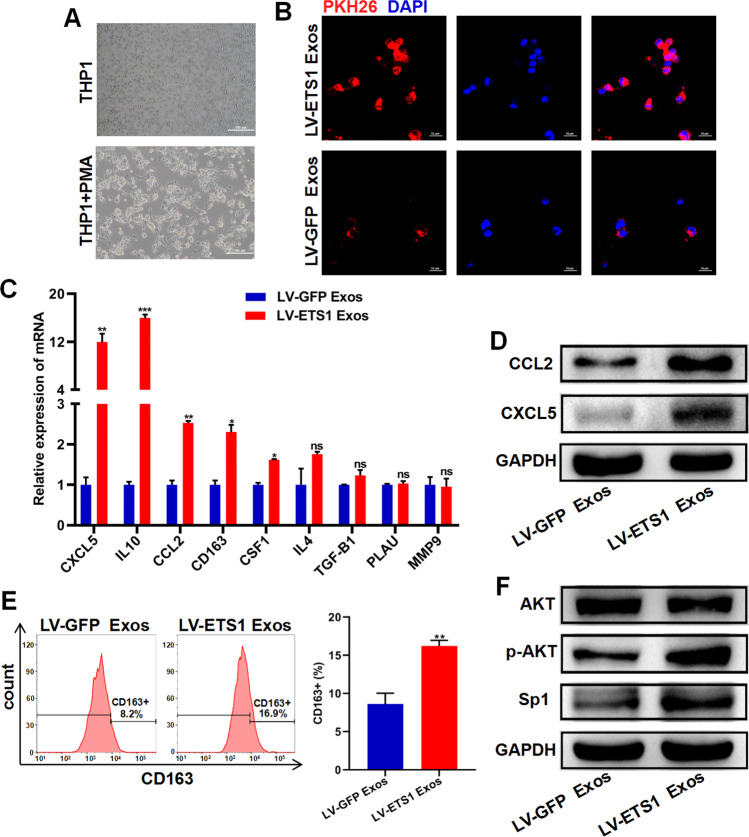
LV-ETS1 Exos induce macrophage polarization toward M2 and increase CXCL5 and CCL2 expression in macrophages. **A** Representative images of THP-1 cells and M0 macrophages derived from THP-1 cells treated with phorbol 12-myristate 13-acetate (PMA) for 24 h. Scale bars: 100 μm at ×100 magnification. **B** Representative immunofluorescence images of M0 macrophages treated with PKH26-labeled LV-ETS1 Exos (red) and LV-GFP Exos (red) for 12 h. Scale bars: 10 μm at ×630 magnification. **C** qRT-PCR analyses showing mRNA levels of M2 macrophage markers and associated cytokines in M0 macrophages treated with LV-ETS1 Exos or LV-GFP Exos for 48 h. **D** Western blotting analyses showing the protein levels of CXCL5 and CCL2 in M0 macrophages treated with LV-ETS1 Exos or LV-GFP Exos for 48 h. **E** Flow cytometry analysis showing the proportion of CD163 + macrophages in M0 macrophages treated with LV-ETS1 Exos or LV-GFP Exos for 48 h. **F** Western blotting analyses showing the protein levels of AKT, p-AKT and Sp1 in M0 macrophages treated with LV-ETS1 Exos or LV-GFP Exos for 48 h. Data are shown as mean ± SD. *P < 0.05, **P < 0.01, ***P < 0.001, ns non-significant.

The human THP-1 cell line was used as a typical mononuclear macrophage cell line. THP-1 cells were treated with PMA to induce their differentiation into M0 macrophages (Fig. 5A). When co-cultured with M0 macrophages, LV-ETS1 Exos and LV-GFP Exos labeled with PKH26 were absorbed by macrophages, and LV-ETS1 Exos were more readily assimilated (Fig. 5B). Upon co-culturing macrophages with either LV-ETS1 Exos or LV-GFP Exos for 48 h, the mRNA levels of the M2 macrophage markers CD163 and IL10 were substantially increased in LV-ETS1 Exos co-cultured macrophages. Notably, CXCL5 and CCL2 mRNAs levels were also considerably higher in the LV-ETS1 Exos group than in the control group, and western blotting confirmed this finding (Fig. 5C, D). Flow cytometry results indicated that LV-ETS1 Exos enhanced the proportion of CD163 + macrophages (Fig. 5E). AKT/Sp1 is a downstream pathway of integrin αvβ5, and Sp1 can regulate the expression of CXCL5 and CCL2 [29–31]. In macrophages co-cultured with LV-ETS1 Exos, we detected activation of the AKT/Sp1 pathway (Fig. 5F). The aforementioned findings show that LV-ETS1 Exos enhance the polarization of macrophages toward the M2 phenotype, as well as the expression of CXCL5 and CCL2 in macrophages, via the integrin αvβ5/AKT/Sp1 signaling pathway.

### LV-ETS1 Exos mediate the pro-tumorigenic effects of macrophages in vitro

**Fig. 6:**
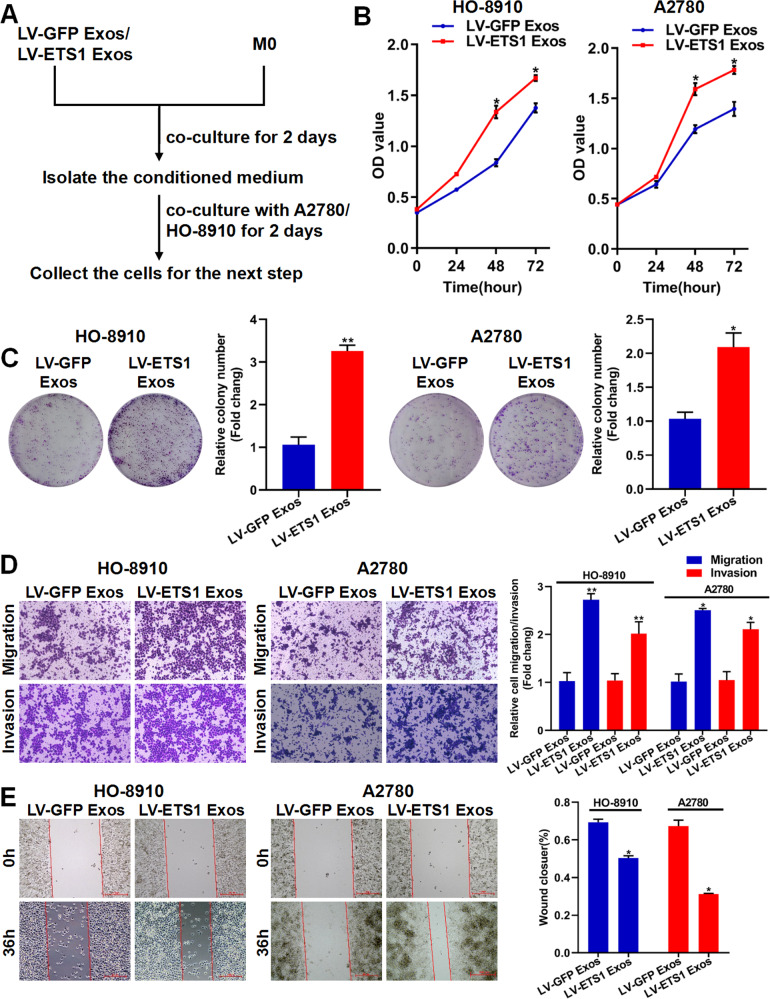
LV-ETS1 Exos promote the proliferation, migration, and invasion of ovarian cancer cells via remodeling macrophages. **A** A sketch illustrating the experimental design. M0 macrophages treated with LV-ETS1 Exos and LV-GFP Exos for 48 h. The effect of the conditioned medium from macrophages treated with exosomes on the proliferation, migration, and invasion of ovarian cancer cells were analyzed by CCK-8 assay, colony-formation assay, Transwell assay and wound-healing assay. **B** The effect of conditioned medium of macrophages treated with LV-ETS1 Exos and LV-GFP Exos on HO-8910 and A2780 cell proliferation was measured by CCK-8 assay. **C** The effect of conditioned medium of macrophages treated with LV-ETS1 Exos and LV-GFP Exos on HO-8910 and A2780 cells proliferation was measured by colony-formation assay. **D** The effect of conditioned medium of macrophages treated with LV-ETS1 Exos and LV-GFP Exos on HO-8910 and A2780 cell migration and invasion were analyzed by Transwell assay. **E** Wound-healing assay showing the horizontal migration ability of ovarian cancer cells co-cultured with conditioned medium of macrophages treated with LV-ETS1 Exos and LV-GFP Exos. Data are shown as mean ± SD. *P < 0.05, **P < 0.01.

We utilized LV-ETS1 Exos and LV-GFP Exos to stimulate M0 macrophages for 48 h and then collected the conditioned medium for co-cultures with ovarian cancer cells for 48 h (Fig. 6A). According to CCK-8 assays and colony-formation experiments, conditioned media from macrophages co-cultured with LV-ETS1 Exos induced a larger increase in HO-8910 and A2780 cell proliferation than those from macrophages treated with LV-GFP Exos (Fig. 6B, C). Transwell experiments showed that LV-ETS1 Exos-treated macrophages considerably increased the invasive and migratory capacities of HO-8910 and A2780 cells (Fig. 6D). Consistent with these findings, conditioned media from macrophages co-cultured with LV-ETS1 Exos strongly stimulated migration, whereas conditioned media from macrophages treated with LV-GFP Exos had a considerably lower impact (Fig. 6E). These findings provide compelling evidence that macrophages exposed to LV-ETS1 Exos enhance the progression of ovarian cancer.

### LV-ETS1 Exos promote omental metastasis of ovarian cancer via integrin αvβ5/AKT/Sp1 signaling

**Fig. 7:**
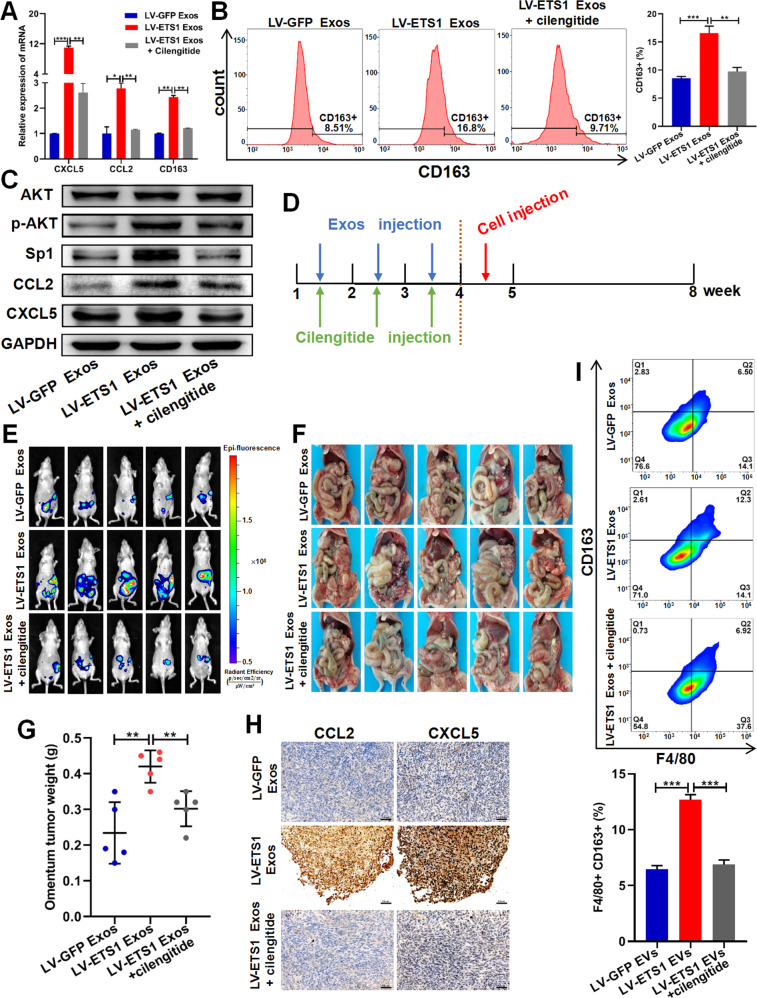
LV-ETS1 Exos promote omentum metastasis of ovarian cancer via integrin αvβ5/AKT/Sp1 signaling. **A** The results of qRT-PCR showing the mRNA levels of CCL2, CXCL5 and CD163 in peritoneal macrophages incubated with LV-ETS1 Exos and cilengitide for 48 h. **B** Flow cytometry analysis showing the proportion of CD163 + macrophages in peritoneal macrophages incubated with LV-ETS1 Exos and cilengitide for 48 h. **C** Western blotting analyses showing the protein levels of CCL2, CXCL5, AKT, p-AKT and Sp1 in peritoneal macrophages incubated with LV-ETS1 Exos and cilengitide for 48 h. **D** Schematic diagram of the experimental schedule. LV-ETS1 Exos and LV-GFP Exos were injected into nude mice every other day, along with cilengitide for three weeks, followed by intraperitoneal injection of A2780-Luc cells. **E** Representative images of metastatic luciferase signal in nude mice from indicated treatment groups at 4 weeks after injection of A2780-Luc cells. **F** Representative images of the omental tumor burden in nude mice from indicated treatment groups at 4 weeks after injection of A2780-Luc cells. **G** Graphical representation of the excised omental tumor weights in nude mice from indicated treatment groups. **H** Representative IHC staining of CCL2 and CXCL5 in omental tumor of indicated treatment groups. Scale bars: 50 μm at ×200 magnification. **I** Flow cytometry analysis showing the proportion of CD163 + macrophages in omental tumor of indicated treatment groups. Data are shown as mean ± SD. *P < 0.05, **P < 0.01, ***P < 0.001.

We isolated mouse peritoneal macrophages and co-incubated them with exosomes and the integrin αvβ5 inhibitor cilengitide to confirm that LV-ETS1 Exos could drive the AKT/Sp1 pathway in macrophages via interaction of upregulated laminins with their receptor integrin αvβ5. Cilengitide treatment reduced LV-ETS1 Exos-mediated CD163, CXCL5, and CCL2 mRNA expression levels in macrophages (Fig. 7A). Flow cytometry data suggested that cilengitide treatment reduced the impact of LV-ETS1 Exos on the M2 polarization of macrophages (Fig. 7B). In addition, cilengitide treatment inhibited the LV-ETS1 Exos-mediated activation of the AKT/Sp1 pathway and downregulated the protein levels of CXCL5 and CCL2 in macrophages (Fig. 7C).

**Fig. 8:**
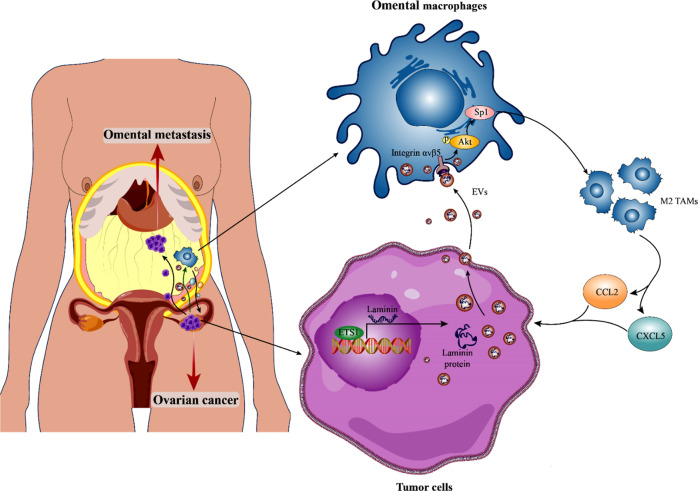
Schematic model showing how ETS1-mediated exosomes secreted by cancer cells regulate ovarian cancer omental metastasis. ETS1 drives ovarian cancer cells to release exosomes with larger sizes and increased laminins levels, hence accelerating the exosomes-mediated pro-tumorigenic effects of omental macrophages via the integrin αvβ5/AKT/Sp1 signaling pathway, consequently promoting omental metastasis of ovarian cancer.

To further evaluate the effects of LV-ETS1 Exos on omental metastasis in ovarian cancer, we established a nude mouse model of omental metastasis in ovarian cancer. We administered LV-GFP Exos and LV-ETS1 Exos every other day together with cilengitide into the peritoneal cavity of nude mice for three weeks, followed by intraperitoneal injection of A2780-Luc cells for experimental modeling (Fig. 7D). Compared to the LV-GFP Exos group, LV-ETS1 Exos boosted ovarian cancer metastasis to the omentum, whereas cilengitide prevented the pro-tumorigenic impact of LV-ETS1 Exos (Fig. 7E–G). The expression of CXCL5 and CCL2 was evaluated by IHC in omental metastases in each group. The findings demonstrated that the expression of CXCL5 and CCL2 was greater in the LV-ETS1 Exos group than in the LV-GFP Exos group and that administration of cilengitide prevented this increase (Fig. 7H). Flow cytometry was used to compare populations of M2 macrophages in omental metastases among groups. Compared to the LV-GFP Exos group and the LV-ETS1 Exos + cilengitide group, the number of F4/80 + CD163 + macrophages in omental metastases was considerably higher in the LV-ETS1 Exos group (Fig. 7I). These findings indicate that LV-ETS1 Exos enhance omental metastasis in ovarian cancer by modulating the pro-tumorigenic effects of macrophages through integrin αvβ5/AKT/Sp1 signaling (Fig. 8).

## Discussion

Ovarian cancer differs from other solid tumors in that ovarian cancer cells disseminate and metastasize mostly inside the peritoneal cavity and are only superficially invasive [32]. During the spread and metastasis of ovarian cancer, cancer cells first separate from the primary site and then implant into the metastatic site, including the omentum, which is the most frequent location of metastasis in ovarian cancer [33]. In the present study, we discovered that ETS1 was highly expressed in ovarian cancer omental metastases and that ETS1 could stimulate the proliferation, invasion, and migration of ovarian cancer cells, indicating that ETS1 was implicated in ovarian cancer omental metastases. Nevertheless, according to an integrated survival analysis of the GEO datasets and the TCGA ovarian cancer cohort, ETS1 mRNA may not be a reliable indicator of ovarian cancer patients. Furthermore, exosomes derived from ETS1-overexpressing ovarian cancer cells mediated pro-tumorigenic effects of omental macrophages via the integrin αvβ5/AKT/Sp1 signaling pathway, consequently promoting omental metastasis of ovarian cancer.

ETS1, the founder of the large ETS transcription factor family, functions primarily as a transcriptional activator and drives key events in advanced cancer progression [34]. According to previous studies, transcription factors can influence the expression of nucleic acids or proteins in tumor cell exosomes, thereby altering tumorigenesis and progression [35–37]. However, the effects of ETS1 on the release and composition of exosomes derived from ovarian cancer cells remain unknown. To our knowledge, this is the first study to demonstrate the function of ETS1-regulated exosomes and exosomal proteins derived from ovarian cancer cells in omental metastasis. ETS1 overexpression altered the size of exosomes derived from ovarian cancer cells, and upregulated the expression of three laminins, LAMA5, LAMB1, and LAMC1, in ovarian cancer cells and their exosomes.

Exosomes are capable of delivering functional biomolecules, such as proteins, lipids, and RNAs, horizontally to recipient cells [38–40]. Exosomes originating from highly metastatic melanoma cells have been demonstrated to educate bone marrow progenitor cells by delivering the MET oncoprotein, enhancing metastatic burden and organotropism [41]. Hoshino et al. discovered that tumor cell-derived exosomal integrins could be transported to organ-specific resident cells to establish a pre-metastatic niche that determines organ-specific metastasis [42]. Nonetheless, the concrete function and mechanism of cancer cells exosomes in ovarian cancer omental metastasis remain unknown. In this study, we discovered that the omentum is the primary intraperitoneal organ for the absorption of ovarian cancer exosomes, and resident macrophages of the omentum were the predominant exosomes receiver cells. Exosomes released from ETS1-overexpressing ovarian cancer cells were specifically attractive to omental macrophages. To elucidate this phenomenon, we investigated the expression of the laminin receptors, integrin family members in the omentum and discovered that integrin αvβ5 had the highest expression level. Intriguingly, integrin αvβ5 has been reported to be highly expressed in macrophages [43–45]. We also found that omental macrophages expressed integrin αvβ5 and that integrin αvβ5-positive cells were receivers of ovarian cancer cell exosomes. We then confirmed that LAMA5, LAMB1, and LAMC1 could bind to integrin αvβ5. Therefore, we concluded that ETS1 enhanced the transport of tumor cell-derived exosomes to omental resident macrophages through exosomal laminins interaction with macrophage integrin αvβ5, laying the foundation for the establishment a pre-metastatic niche.

Ovarian cancer cells initially metastasize to milky spots in the omentum but not to other intra-abdominal tissues, a process that is independent of the presence of B cells, T cells, or NK cells in the omentum; however, omentum-resident macrophages promote early metastatic colonization of the omentum [46]. Omental macrophage density increases proportionally with the omental disease score in patients with ovarian cancer [47]. We found that elevated laminin levels in LV-ETS1 Exos activated the AKT/Sp1 pathway in macrophages by interacting with integrin αvβ5 to upregulate the expression of CXCL5 and CCL2 in macrophages and increase the proportion of CD163 + macrophages. CXCL5 and CCL2 released by macrophages in the tumor microenvironment can enhance tumor metastasis by interacting with their respective receptors in tumor cells [48, 49]. In ovarian cancer, both CXCL5 and CCL2 are pro-metastatic cytokines [50, 51]. Increased CD163 expression on tumor-associated macrophages (TAMs) correlates with poor clinical outcomes in different malignancies [52]. Depletion of CD163 + macrophages can restrict omental metastasis of ovarian cancer [53]. In vitro, the conditioned medium of LV-ETS1 Exos co-cultured with macrophages could upregulate ovarian cancer proliferation, migration, and invasion, compared with that of LV-GFP Exos. In vivo experiments also confirmed that LV-ETS1 Exos could mediate the pro-tumorigenic impact of omental macrophages and promote the metastasis and colonization of ovarian cancer cells to the omentum, Next, we utilized cilengitide to further clarify whether macrophage function was regulated by laminins and integrin αvβ5 interactions. Cilengitide is an inhibitor that competitively antagonizes the integrin αvβ3 and αvβ5 receptors, presently being tested in preclinical cancer treatment research [54]. The results showed that cilengitide inhibited laminins-integrin αvβ5-mediated activation of AKT/Sp1 pathway and reversed LV-ETS1 Exos-mediated macrophage pro-tumorigenic effects.

Taken together, our findings indicate that the interaction of omental macrophages with ETS1-regulated exosomes affects omental metastasis in ovarian cancer. ETS1 can drive ovarian cancer cells to release exosomes with higher laminin levels, thereby accelerating the exosomes-mediated pro-metastatic effects of omental macrophages via the integrin αvβ5/AKT/Sp1 signaling pathway, and cilengitide can inhibit omental metastasis of ovarian cancer driven by tumor-derived exosomes. Our results demonstrate the important role of laminins in exosomes generated from ETS1-overexpressing ovarian cancer cells in tumor progression by remodeling the tumor microenvironment and provide novel insights that may be useful in the treatment of omental metastasis in ovarian cancer.

## Supplementary information

Supplementary tables and figures

Original western blots

Reproducibility Checklist

## Data Availability

The datasets obtained and/or analyzed during this study are available from the corresponding authors in a reasonable request.
