# Supplementary material for: Laminins in tumor-derived exosomes upregulated by ETS1 reprogram omental macrophages to promote omental metastasis of ovarian cancer
Source: Cell Death Dis. 2022 Dec 7;13(12):1028. doi: 10.1038/s41419-022-05472-7 (PMC9729302; doi:10.1038/s41419-022-05472-7)
Supplement: Supplementary file 1 — Supplementary tables and figures [file 41419_2022_5472_MOESM1_ESM.docx]

**Table S1.** Sequences of forward and reverse primers used for qRT-PCR and ChIP-qRCR.

| Species | Gene | Forward Primer | Reverse Primer |
| --- | --- | --- | --- |
| Homo | ETS1 | GATAGTTGTGATCGCCTCACC | GTCCTCTGAGTCGAAGCTGTC |
| Homo | LAMA5 | CCCACCGAGGACCTTTACTG | GGTGTGCCTTGTTGCTGTT |
| Homo | LAMB1 | CACAAGCCCGAACCCTACTG | GACCACATTTTCAATGAGATGGC |
| Homo | LAMC1 | ACTGCCACTGACATCAGAGTA | GCTTGCGTGTCCATTACATTTAC |
| Homo | CCL2 | CAGCCAGATGCAATCAATGCC | TGGAATCCTGAACCCACTTCT |
| Homo | CXCL5 | CTGCGTTGCGTTTGTTTAC | GGCTACCACTTCCACCTTG |
| Homo | CD163 | TTTGTCAACTTGAGTCCCTTCAC | TCCCGCTACACTTGTTTTCAC |
| Homo | IL10 | GACTTTAAGGGTTACCTGGGTTG | TCACATGCGCCTTGATGTCTG |
| Homo | MMP9 | TGTACCGCTATGGTTACACTCG | GGCAGGGACAGTTGCTTCT |
| Homo | PLAU | GGGAATGGTCACTTTTACCGAG | GGGCATGGTACGTTTGCTG |
| Homo | GAPDH | GGAGCGAGATCCCTCCAAAAT | GGCTGTTGTCATACTTCTCATGG |
| Homo | CSF1 | TGGCGAGCAGGAGTATCAC | AGGTCTCCATCTGACTGTCAAT |
| Homo | IL4 | CCAACTGCTTCCCCCTCTG | TCTGTTACGGTCAACTCGGTG |
| Homo | TGFB1 | GGCCAGATCCTGTCCAAGC | GTGGGTTTCCACCATTAGCAC |
| Mus | CCL2 | TAAAAACCTGGATCGGAACCAAA | GCATTAGCTTCAGATTTACGGGT |
| Mus | CXCL5 | GTTCCATCTCGCCATTCATGC | GCGGCTATGACTGAGGAAGG |
| Mus | CD163 | GGTGGACACAGAATGGTTCTTC | CCAGGAGCGTTAGTGACAGC |
| Mus | GAPDH | AGGTCGGTGTGAACGGATTTG | GGGGTCGTTGATGGCAACA |
| Homo | LAMA5(Chip) | CCACTTGGAGCTTCTCCTGC | TTCTCTACGGAGTCGGGAGG |
| Homo | LAMB1  (Chip) | CCTCACTCACCTAGACCCCAA | CCACCCATTCTGCTACCCTT |
| Homo | LAMC1  (Chip) | CCCACTGGCTGGTTACACTTT | TTGCTGCCTTTTGAGTCCTAAT |

**Table S2.** Antibody used for for western blotting (WB), immunoprecipitation (IP), immunohistochemical (IHC) and immunofluorescence (IF).

| Name | Brand | Cat. No. | Dilution |
| --- | --- | --- | --- |
| ETS1 Antibody | Cusabio | CSB-PA008154 | 1:1000(WB)  1:200(IHC)  1:200(IF) |
| GAPDH antibody | Proteintech | 10494-1-AP | 1:20000(WB) |
| CD63 Antibody | Santa Cruz | sc-5275 | 1:200(WB) |
| CD81 Antibody | Santa Cruz | sc-166029 | 1:100(WB) |
| Tsg101 Antibody | Santa Cruz | sc-7964 | 1:200(WB) |
| Alix Antibody | Santa Cruz | sc-53540 | 1:200(WB) |
| Anti-Hsp70 Rabbit | Servicebio | GB11241 | 1:800(WB) |
| LAMA5 Antibody | Cusabio | CSB-PA009767 | 1:1000(WB)  1:200(IF) |
| LAMB1 Antibody | Cusabio | CSB-PA003135 | 1:1000(WB)  1:200(IF) |
| LAMC1 Antibody | Cusabio | CSB-PA009776 | 1:1000(WB)  1:200(IF) |
| Integrin β5/ITGB5 Antibody | Santa Cruz | sc-398214 | 1:100(WB)  2µg/400µg total protein (IP)  1:50(IF)  1:50(IHC) |
| Anti-Integrin alpha V Antibody | Abcam | ab179475 | 1:5000(WB)  1:40(IP)  1:500(IF)  1:500(IHC) |
| CXCL5 Antibody | Affinity | DF9919 | 1:2000(WB)  1:100(IHC) |
| MCP1 (CCL2) Antibody | Affinity | DF7577 | 1:2000(WB)  1:200(IHC) |
| AKT antibody | Proteintech | 60203-2-Ig | 1:10000(WB) |
| Phospho-AKT (Ser473) antibody | Proteintech | 28731-1-AP | 1:3000(WB) |
| SP1 antibody | Proteintech | 21962-1-AP | 1:3000(WB) |
| HRP-conjugated Affinipure Goat Anti-Rabbit IgG(H+L) | Proteintech | SA00001-2 | 1:5000(WB) |
| HRP-conjugated Affinipure Goat Anti-Mouse IgG(H+L) | Proteintech | SA00001-1 | 1:5000(WB) |
| CoraLite488-conjugated Goat Anti-Rabbit IgG(H+L) | Proteintech | SA00013-2 | 1:200(IF) |
| CoraLite594-conjugated Goat Anti-Rabbit IgG(H+L) | Proteintech | SA00013-4 | 1:200(IF) |

**Table S3.** Antibody used for flow cytometry.

| Name | Brand | Cat. No. |
| --- | --- | --- |
| PE anti-human CD163 Antibody | BioLegend | 33605 |
| anti-mouse F4/80 Antibody (BM8), PE | eBioscience | 12-4801-82 |
| anti-mouse CD163 Antibody (TNKUPJ), APC | eBioscience | 17-1631-82 |
| PerCP/Cyanine5.5 anti-mouse/human CD11b Antibody | BioLegend | 101228 |
| anti-mouse CD45 Antibody (30-F11), PE-Cyanine7 | eBioscience | 25-0451-82 |

**Supplementary figure**

**
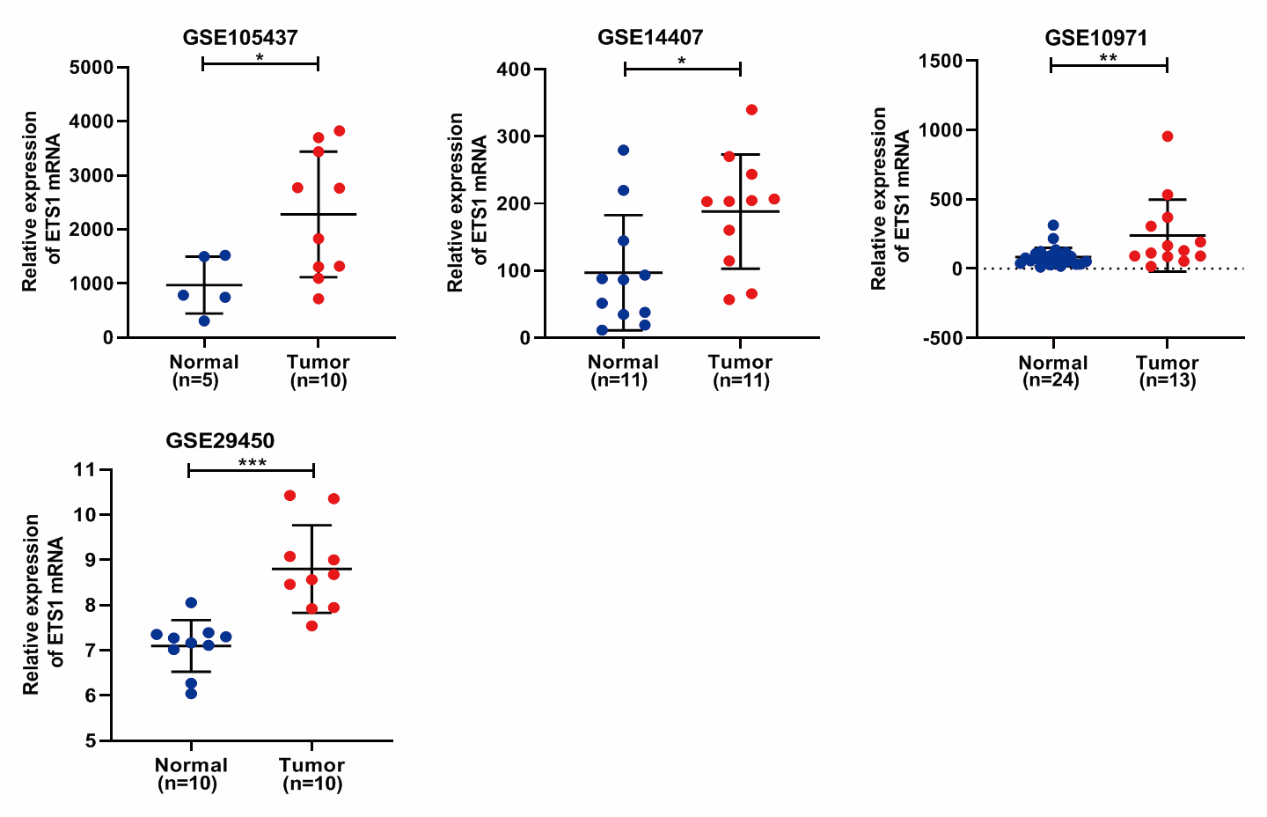
**

**Figure S1. Expression of ETS1 mRNA in the ovarian cancer tissues and normal tissues from the GSE105437, GSE14407, GSE10971 and GSE29450 datasets.** Data are shown as mean ± SD. **P* < 0.05, ***P* < 0.01, ****P* < 0.001.


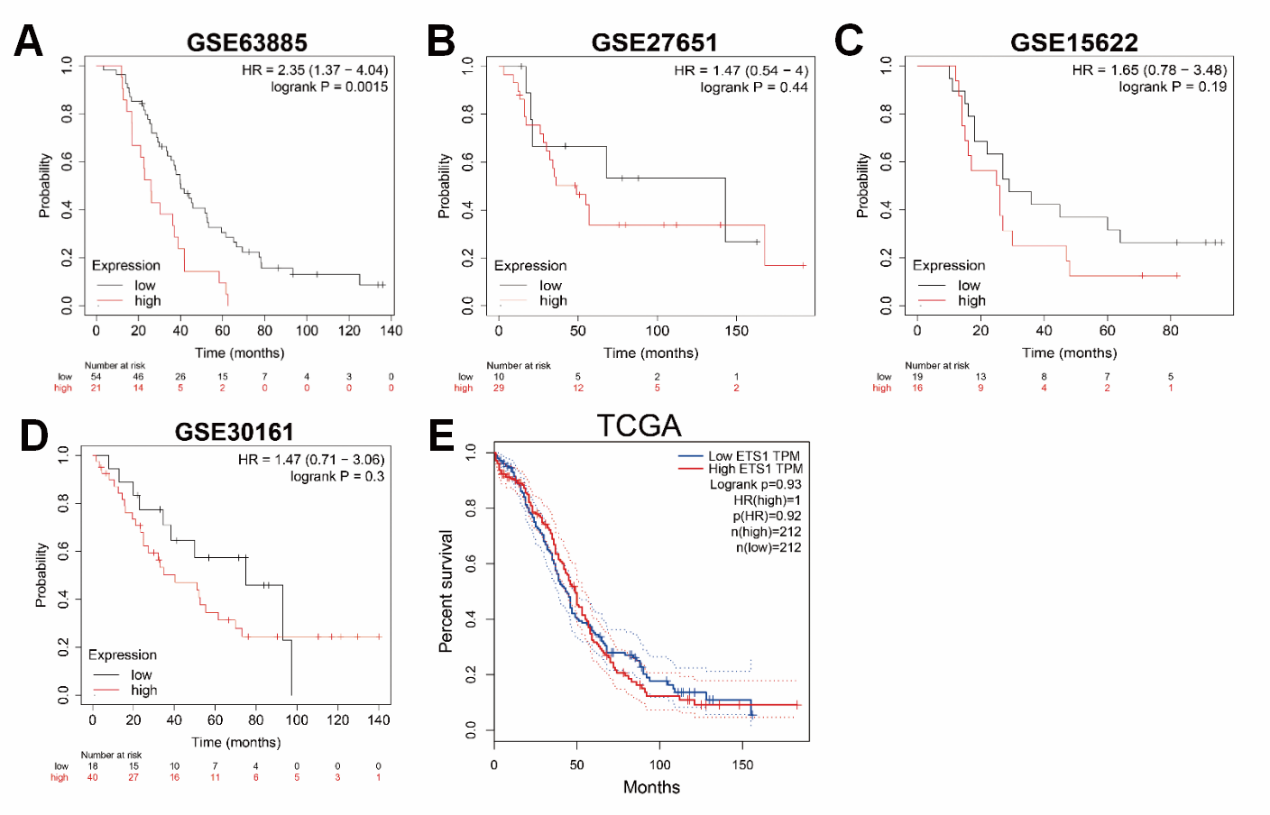


**Figure S2. Relationship between ovarian cancer patients' overall survival and ETS1 mRNA expression across different datasets. (A-E)** Kaplan-Meier plots showing the overall survival of ovarian cancer patients from the GSE63885 **(A)**, GSE27651 **(B)**, GSE15622 **(C)**, GSE30161 **(D)** and TCGA ovarian cancer cohorts **(E)** based on ETS1 mRNA expression.

**
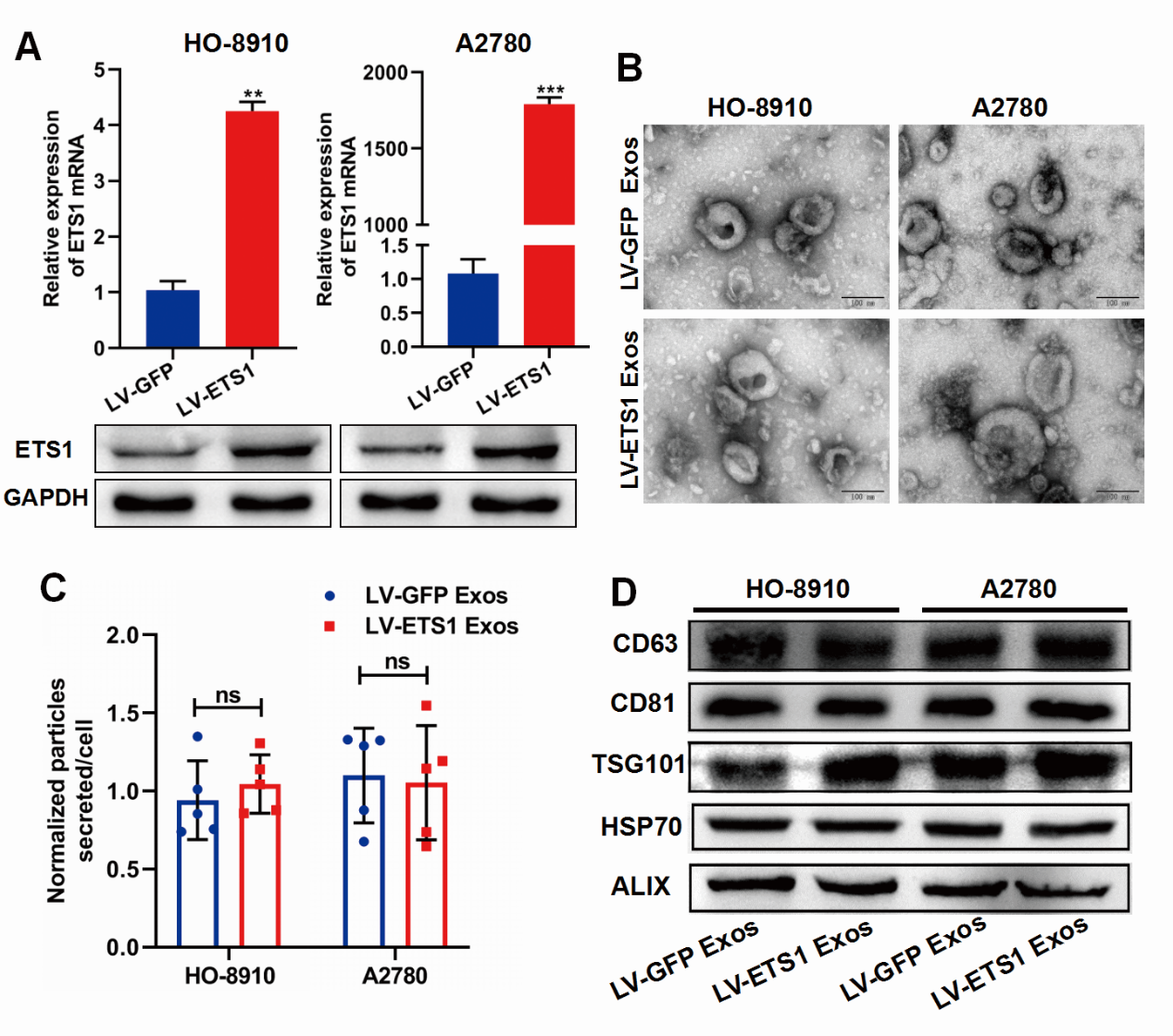
**

**Figure S3. Characterization of ovarian cancer cells exosomes.** (**A**) Relative mRNA and protein levels of ETS1 in ovarian cancer cells transfected with ETS1-overexpressing lentivirus detected by qRT-PCR and western blotting. (**B**) Representative transmission electron microscopy (TEM) images of exosomes derived from ovarian cancer cells transfected with lentivirus LV-ETS1 and LV-GFP (LV-ETS1 Exos and LV-GFP Exos). (**C**) Exosomes secretion per cell normalized to the LV-GFP Exos average for each cell. (**D**) Western blotting of exosome marker proteins in LV-ETS1 Exos and LV-GFP Exos. Data are shown as mean ± SD. ***P* < 0.01, ****P* < 0.001, ns, non-significant.


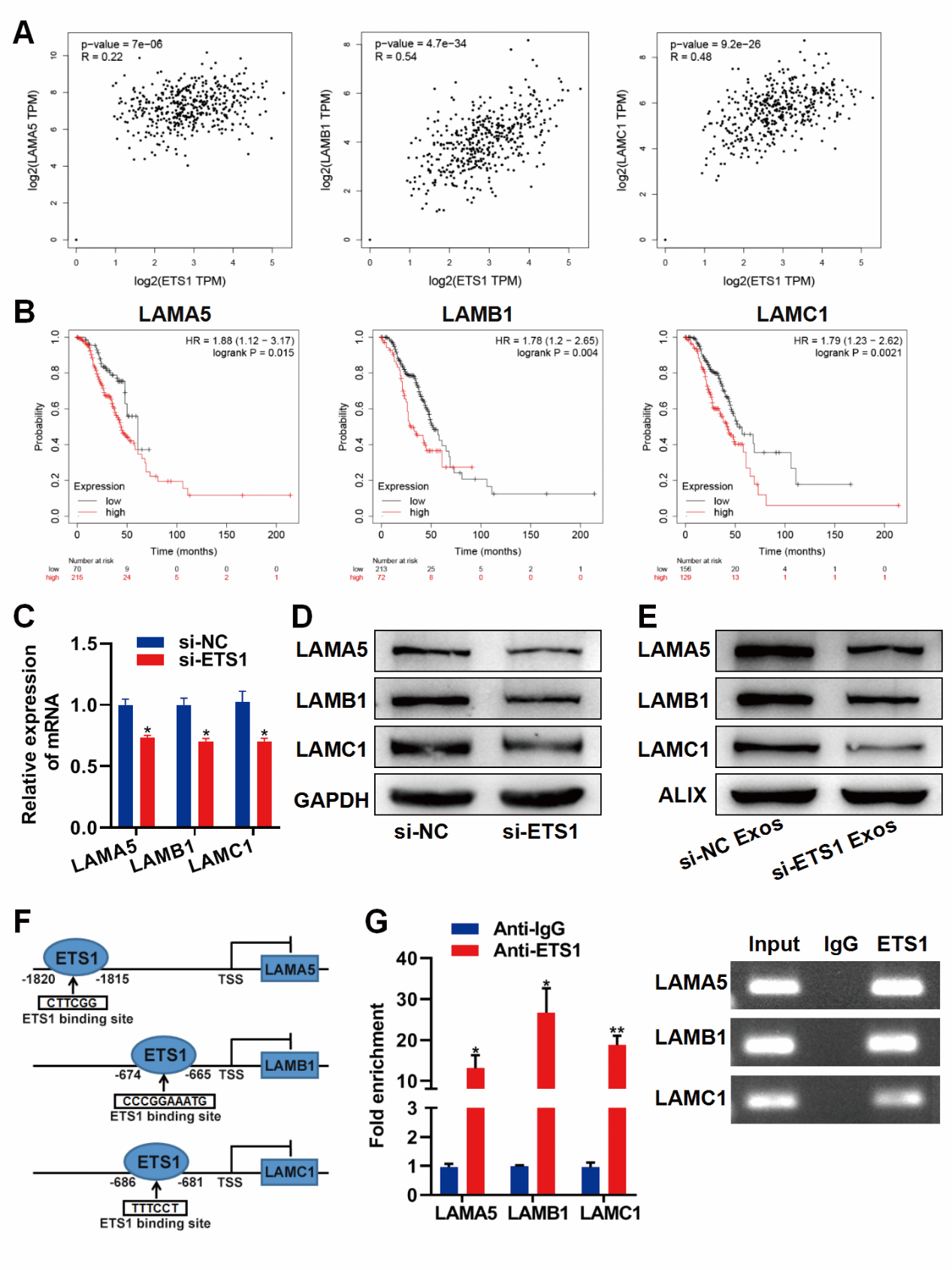


**Figure S4. ETS1 transcriptionally regulates the expression of LAMA5, LAMB1 and LAMC1 in ovarian cancer cells.** (**A**) Correlation analysis showing the correlation of ETS1 mRNA levels with LAMA5, LAMB1 and LAMC1 mRNA levels from TCGA ovarian cancer cohorts. (**B**) Kaplan-Meier plots showing the overall survival of ovarian cancer patients from the GSE9891 database based on LAMA5, LAMB1 and LAMC1 mRNA expression. (**C**) Relative mRNA levels of LAMA5, LAMB1 and LAMC1 in HO-8910 cells after si-ETS1 and si-NC treatment detected by qRT-PCR. (**D**) Western blotting showing LAMA5, LAMB1 and LAMC1 protein expression in ovarian cancer cells transfected with si-ETS1 and si-NC. (**E**) Western blotting showing LAMA5, LAMB1 and LAMC1 protein expression in exosomes derived from ovarian cancer cells transfected with si-ETS1 and si-NC (si-ETS1 Exos and si-NC Exos). (**F**) Schematic diagrams showing the putative binding sites for ETS1 in the promoter regions of LAMA5, LAMB1, and LAMC1 using the Jaspar^2022^ database. (**G**) Enrichment of ETS1 on the LAMA5, LAMB1, and LAMC1 promoter detected by ChIP-qPCR (Left). The ChIP-qPCR products were separated by 2% agarose gel electrophoresis (Right). Data are shown as mean ± SD. **P* < 0.05, ***P* < 0.01.


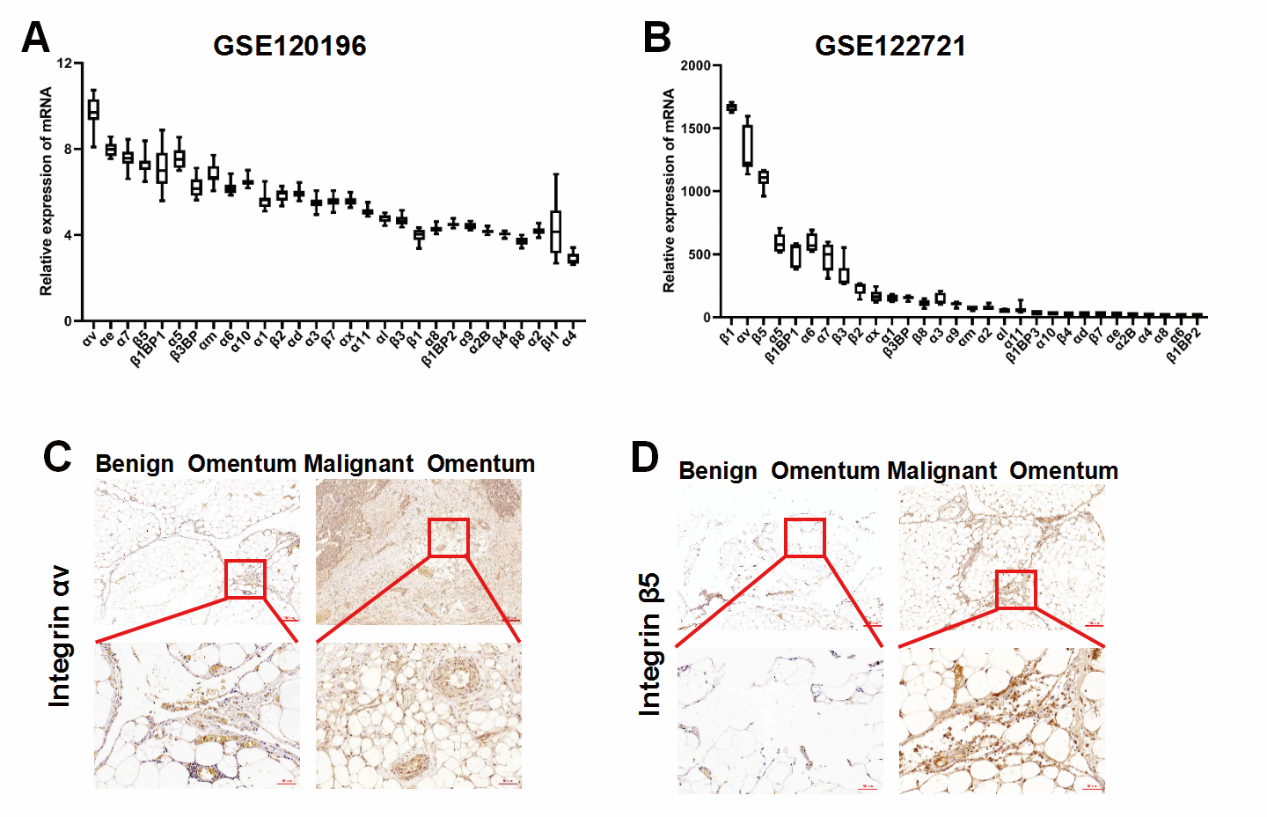


**Figure S5. Expression of integrin αv and integrin β5 in omentum.** (**A-B**) Boxlpots showing integrin family genes mRNA expression of the omentum by integrating the omentum tissue-related GSE120196 (**A**) and GSE122721 (**B**) datasets. (**C-D**) Representative images of integrin αv (**C**) and integrin β5 (**D**) staining in benign omentum and malignant omentum. Scale bars: 200 μm at 40× magnification; 50 μm at 200× magnification. Data are shown as mean ± SD.
