# Supplementary material for: Laminins in tumor-derived exosomes upregulated by ETS1 reprogram omental macrophages to promote omental metastasis of ovarian cancer
Source: Cell Death Dis. 2022 Dec 7;13(12):1028. doi: 10.1038/s41419-022-05472-7 (PMC9729302; doi:10.1038/s41419-022-05472-7)
Supplement: Supplementary file 2 — Original western blots [file 41419_2022_5472_MOESM2_ESM.pptx]

## Slide 1
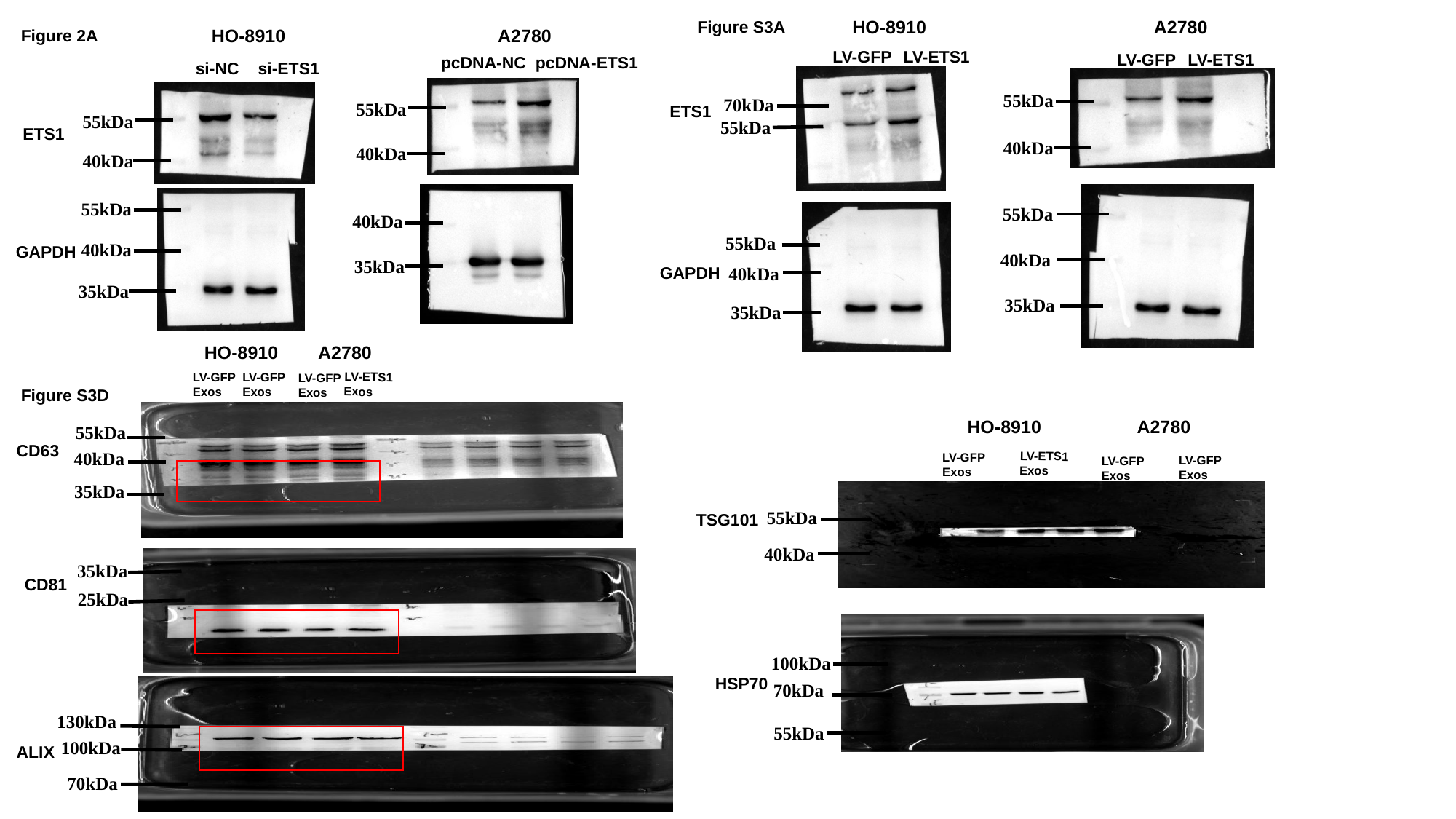

HO-8910 A2780
 Figure S3A
 HO-8910 A2780
 pcDNA-NC pcDNA-ETS1
 si-NC si-ETS1
55kDa
55kDa
 ETS1
40kDa
40kDa
55kDa
40kDa
40kDa
GAPDH
35kDa
35kDa
 Figure 2A
 LV-GFP LV-ETS1
 LV-GFP LV-ETS1
55kDa
70kDa
 ETS1
55kDa
40kDa
55kDa
55kDa
40kDa
40kDa
GAPDH
35kDa
35kDa
 HO-8910 A2780
 LV-ETS1
 Exos
 LV-GFP
 Exos
 LV-GFP
 Exos
 Figure S3D
55kDa
CD63
40kDa
35kDa
35kDa
CD81
 25kDa
130kDa
100kDa
ALIX
70kDa
 LV-GFP
 Exos
 HO-8910 A2780
 LV-ETS1
 Exos
 LV-GFP
 Exos
 LV-GFP
 Exos
55kDa
TSG101
40kDa
100kDa
HSP70
70kDa
55kDa
 LV-GFP
 Exos

## Slide 2
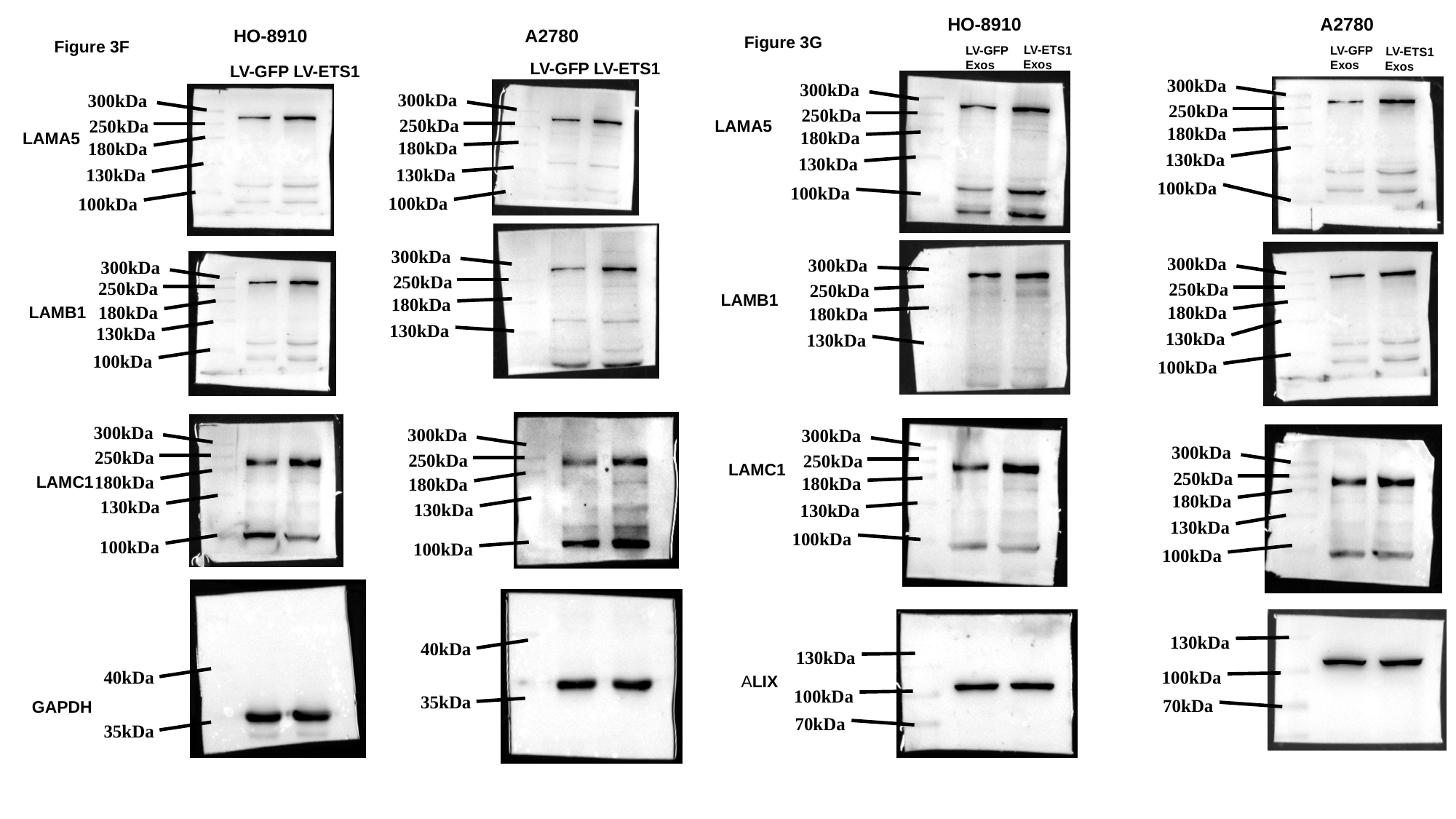

HO-8910 A2780
 HO-8910 A2780
 Figure 3G
 Figure 3F
 LV-ETS1
 Exos
 LV-GFP
 Exos
 LV-GFP
 Exos
 LV-ETS1
 Exos
 LV-GFP LV-ETS1
 LV-GFP LV-ETS1
300kDa
300kDa
300kDa
300kDa
 LAMA5
250kDa
250kDa
 LAMA5
250kDa
250kDa
180kDa
180kDa
180kDa
180kDa
130kDa
130kDa
130kDa
130kDa
100kDa
100kDa
100kDa
100kDa
300kDa
300kDa
300kDa
300kDa
 LAMB1
250kDa
250kDa
250kDa
250kDa
 LAMB1
180kDa
180kDa
180kDa
180kDa
130kDa
130kDa
130kDa
130kDa
100kDa
100kDa
300kDa
300kDa
300kDa
300kDa
250kDa
250kDa
250kDa
 LAMC1
250kDa
180kDa
 LAMC1
180kDa
180kDa
180kDa
130kDa
130kDa
130kDa
130kDa
100kDa
100kDa
100kDa
100kDa
130kDa
40kDa
130kDa
40kDa
100kDa
 ALIX
 GAPDH
100kDa
35kDa
 70kDa
 70kDa
35kDa

## Slide 3
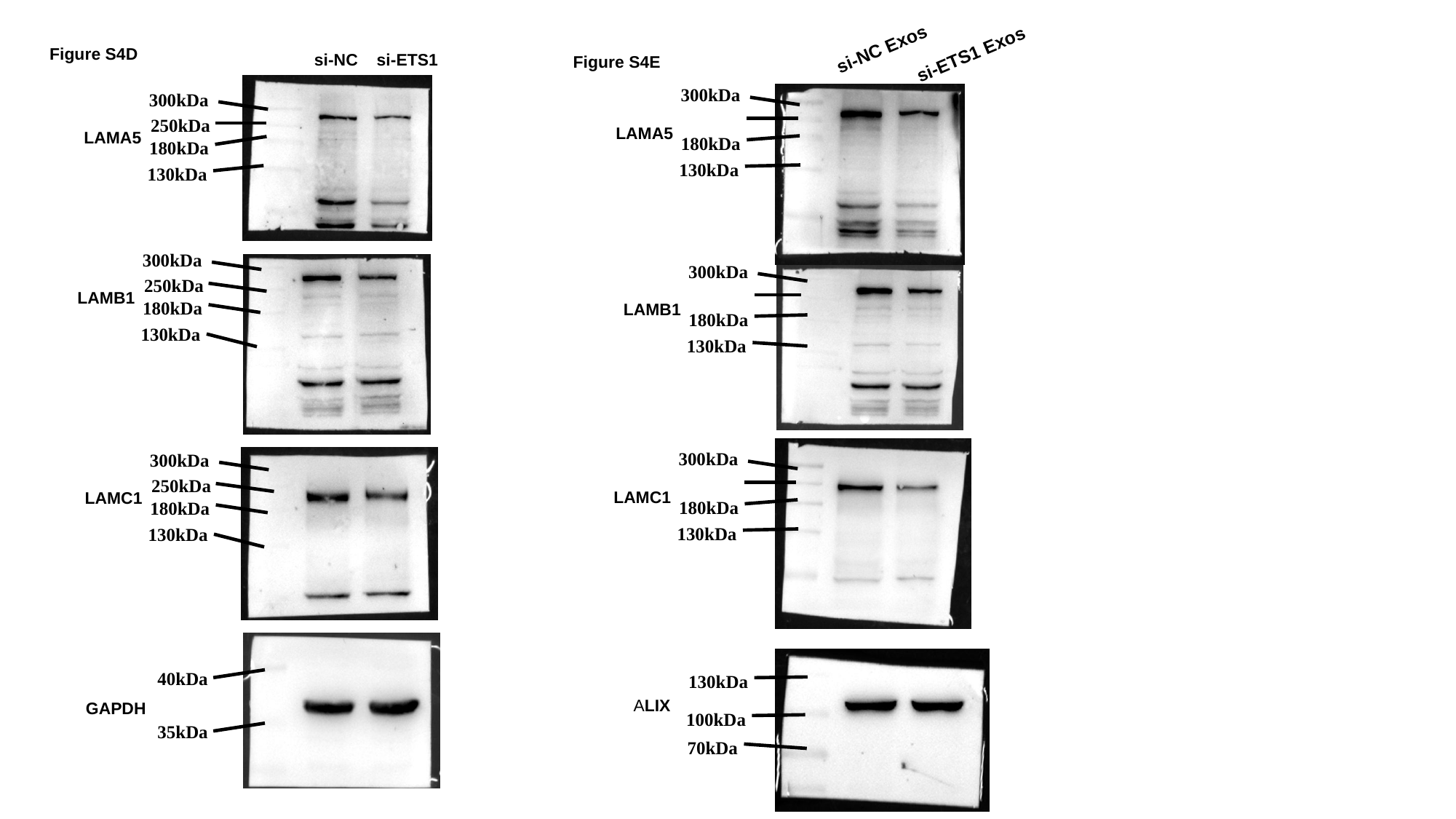

si-NC Exos
si-ETS1 Exos
 Figure S4D
 si-NC si-ETS1
 Figure S4E
300kDa
300kDa
 LAMA5
 LAMA5
250kDa
180kDa
180kDa
130kDa
130kDa
300kDa
300kDa
 LAMB1
250kDa
 LAMB1
180kDa
180kDa
130kDa
130kDa
300kDa
300kDa
 LAMC1
 LAMC1
250kDa
180kDa
180kDa
130kDa
130kDa
40kDa
130kDa
 GAPDH
 ALIX
100kDa
35kDa
 70kDa

## Slide 4
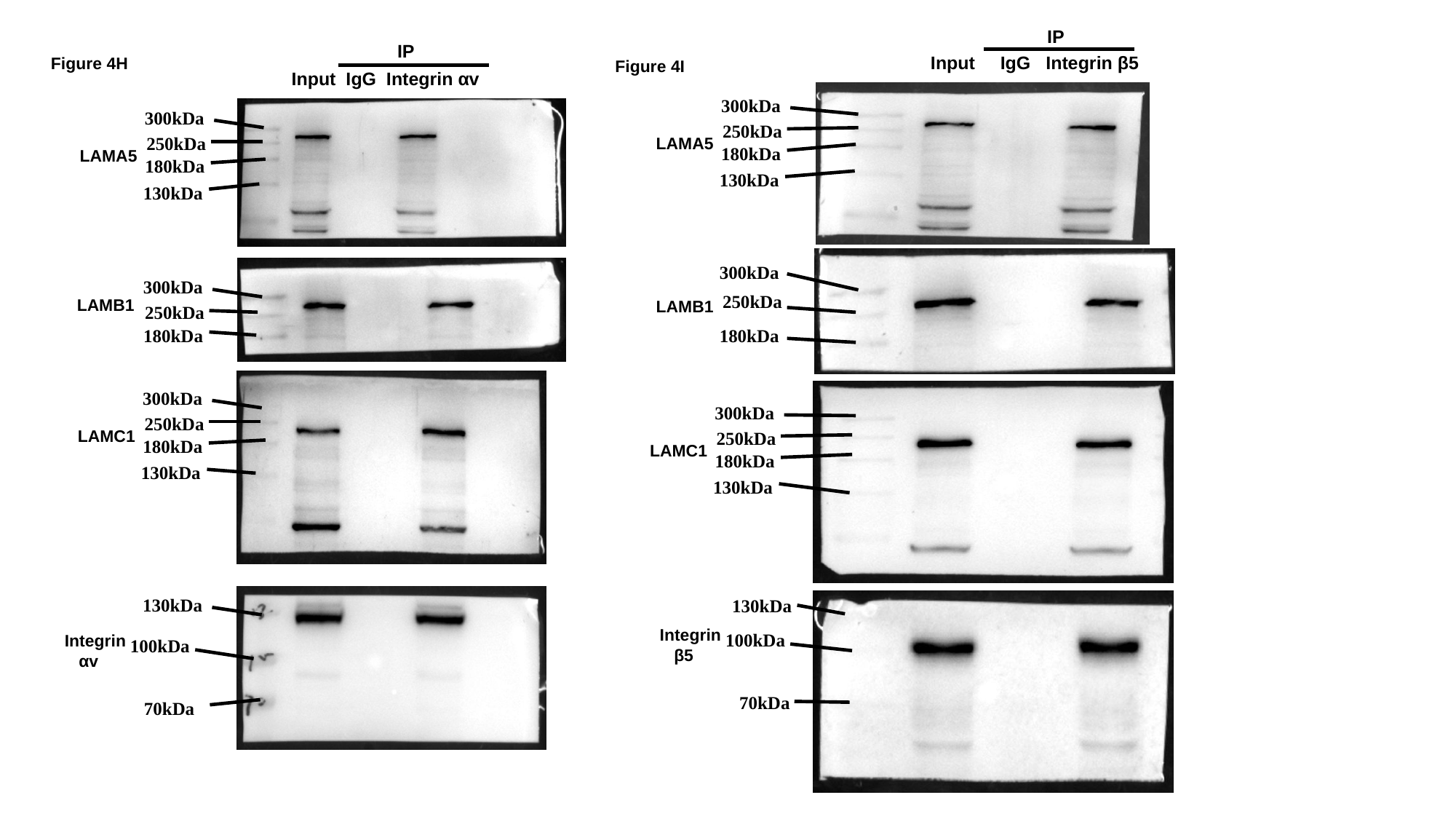

IP
 IP
 Input IgG Integrin β5
 Figure 4H
 Figure 4I
 Input IgG Integrin αv
300kDa
300kDa
 LAMA5
250kDa
 LAMA5
250kDa
180kDa
180kDa
130kDa
130kDa
300kDa
 LAMB1
300kDa
 LAMB1
250kDa
250kDa
180kDa
180kDa
300kDa
300kDa
 LAMC1
250kDa
 LAMC1
250kDa
180kDa
180kDa
130kDa
130kDa
130kDa
130kDa
 Integrin
 β5
100kDa
 Integrin
 αv
100kDa
70kDa
70kDa

## Slide 5
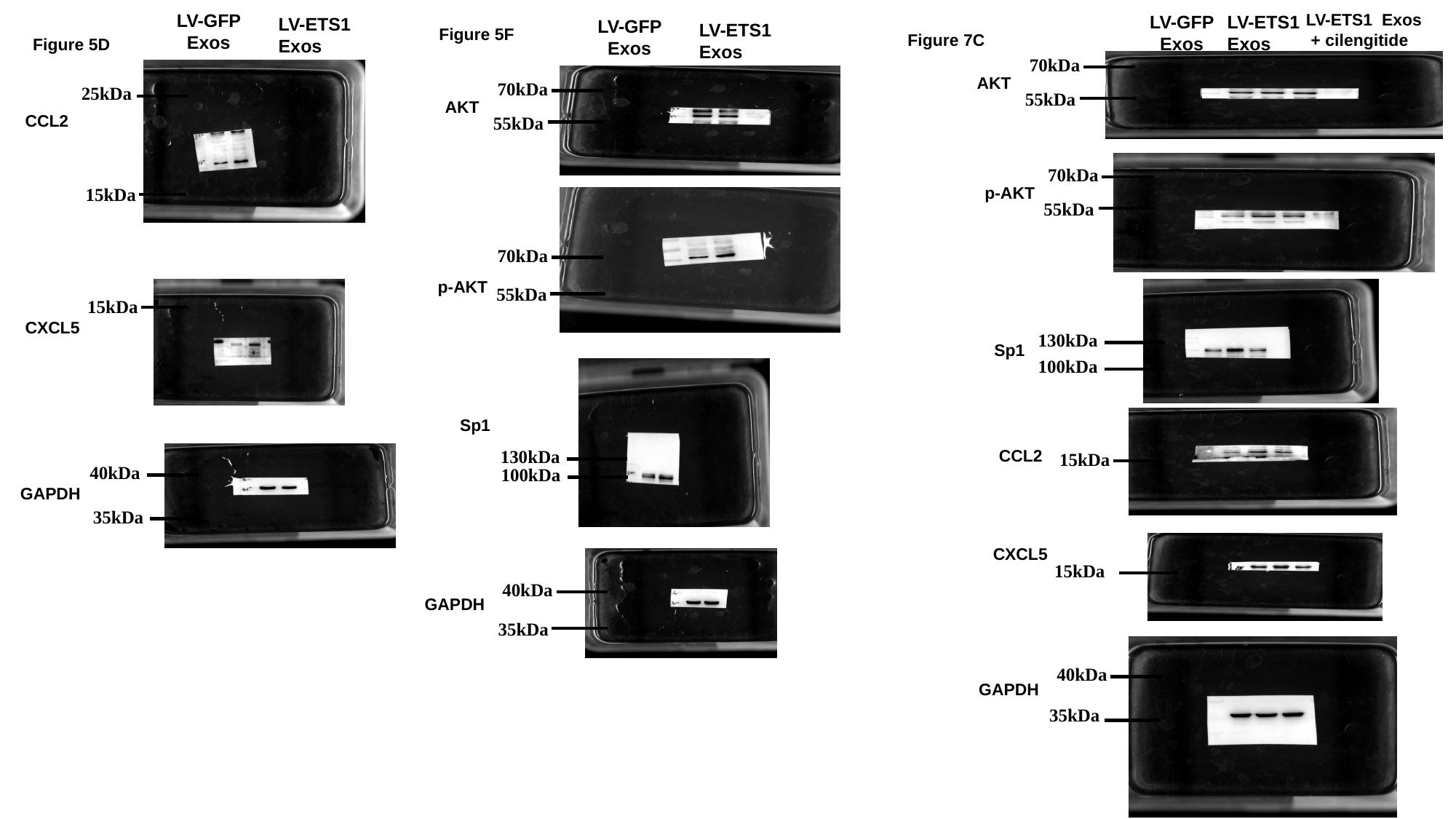

LV-GFP
 Exos
LV-ETS1 Exos
 + cilengitide
LV-GFP
 Exos
LV-ETS1
Exos
LV-ETS1
Exos
LV-GFP
 Exos
LV-ETS1
Exos
 Figure 5F
 Figure 7C
 Figure 5D
 70kDa
AKT
 70kDa
 25kDa
55kDa
AKT
 CCL2
55kDa
 70kDa
p-AKT
15kDa
55kDa
 70kDa
p-AKT
55kDa
15kDa
 CXCL5
130kDa
Sp1
100kDa
Sp1
130kDa
 CCL2
15kDa
 40kDa
100kDa
GAPDH
35kDa
 CXCL5
15kDa
 40kDa
GAPDH
35kDa
 40kDa
GAPDH
35kDa
